# Synergistic effect of transporter and pathway engineering on the key performance indicators of erythritol synthesis by the yeast *Yarrowia lipolytica*

**DOI:** 10.1128/aem.00061-25

**Published:** 2025-03-26

**Authors:** Shuo Xu, Qian Li, Ye Li, Yue Zhang, Qing Li, Liyun Ji, Hairong Cheng

**Affiliations:** 1 State Key Laboratory of Microbial Metabolism and School of Life Sciences and Biotechnology, Shanghai Jiao Tong University 553742 , Shanghai, China; Danmarks Tekniske Universitet The Novo Nordisk Foundation Center for Biosustainability, Kgs. Lyngby, Denmark

**Keywords:** pathway engineering, glucose transporter, erythritol, *Yarrowia lipolytica*

## Abstract

Erythritol, a food additive, is produced on an industrial scale using the yeast *Yarrowia lipolytica*. Nevertheless, the key performance indicators (KPIs) have been found to be unsatisfactory, resulting in elevated erythritol production cost. This study demonstrated that the KPIs (titer, productivity, and yield) of erythritol can be improved by the synergistic application of transporter and pathway engineering strategies in the producing strain. The engineered *Y. lipolytica* strain Ylxs48 exhibits a glucose consumption rate of 310 g/L of glucose within 46 h during batch culture in 3, 100, and 200 L bioreactors as compared to above 72 h for the parental strain Ylxs01. The erythritol yield achieved ranges from 0.69 to 0.74 g/g depending on the culture conditions as compared to 0.55–0.57 g/g for the parental strain Ylxs01. The productivity surpasses 4.60 g/(L·h), representing a 1.91-fold improvement over the parental strain Ylxs01 in 3, 100, or 200 L bioreactors. Under fed-batch conditions in a 200 L bioreactor, an erythritol titer of 355.81 g/L was achieved, marking the highest titer ever reported. This increased erythritol titer enabled crystallization at 4°C directly from the clear supernatant, eliminating the requirement for evaporation or concentration steps. A comprehensive techno-economic analysis of the entire process conclusively demonstrated that implementing the industrial process based on the engineered strain Ylxs48 led to a significant 23% reduction in production cost. This approach holds the potential to substantially reduce erythritol costs and provides novel insights for engineering other industrial strains.

**IMPORTANCE:**

The expansion of the erythritol market attracted excessive capital injection, resulting in overcapacity, operational difficulties, and even bankruptcy of erythritol manufacturers. Technology upgrades in the industry are imminent. However, the production technology of existing enterprises is seriously homogenized, and there is a lack of competitive core-producing strains. In this study, the industrial erythritol-producing strain *Y. lipolytica* CGMCC7326 was genetically modified by integrating substrate transport and pathway modification, which improved the conversion of glucose and significantly improved KPIs, thereby reducing the erythritol production cost.

## INTRODUCTION

The worldwide obesity epidemic has boosted the sugar substitute industry (1). Among the various sugar substitute products, erythritol, a natural sweetener, has been widely used in the food industry because of its unique properties, such as higher daily acceptable intake and safety, low moisture absorption, and no aftertaste compared with other sugar alcohol sweeteners (2). The chemical synthesis of erythritol is unfavorable because of its low yield, high complexity, and harsh operating conditions (3). Consequently, erythritol is the sole sugar alcohol for commercialization through the fermentation process. Erythritol is derived from glucose fermentation by hyperosmotic yeast and was first developed and mass-produced by *Aureobasidium* sp. in Japan (4, 5). Until 2015, the methods for optimizing the erythritol production involved strain screening and mutagenesis breeding, optimization of the medium, and culture mode (6, 7). The titer, yield, and productivity of erythritol ranged from 46 to 243 g/L, 0.26–0.67 g/g, and 0.19–2.84 g/(L⋅h), respectively.

Among the many erythritol-producing yeasts, *Yarrowia lipolytica* has garnered increasing attention because of its excellent gene operability, extensive substrate utilization, and high erythritol production (8–10). After mutagenesis and optimization of culture conditions, the maximum titer, yield, and productivity of erythritol by *Y. lipolytica* can reach 231.2 g/L (11), 0.67 g/g (12), and 1.56 g/(L⋅h) (13), respectively. Although the titer and yield of erythritol have reached an industrial level, the production efficiency (productivity) remains relatively low compared with other yeasts [2.84 g/(L⋅h) by *Pseudozyma tsukubaensis*], which means that the production cost increases owing to longer fermentation times (14). Hence, it is imperative to enhance the conversion efficiency by utilizing metabolic engineering techniques. Mirończuk et al. first applied glycerol-to-erythritol metabolic engineering to modify *Y. lipolytica* to accelerate erythritol production by glycerol utilization. The erythritol productivity for glycerol kinase (GK or GUT1) overexpression and co-expression of GK and glycerol-3-phosphate dehydrogenase (GDH or GUT2) increased by 24% and 35%, respectively, compared with the control strain (15). Subsequently, various laboratories conducted numerous related studies and achieved impressive research outcomes (16). Nevertheless, most of this research has focused on modifying the synthetic pathway using glycerol as the carbon source (17, 18). Crude glycerol is a cost-effective carbon source that can be used to produce various chemical products. Erythritol is a potential platform compound that may be used in large quantities in the chemical industry in the future (18–20). However, at the current time, erythritol is mainly used in the food industry, which has high requirements for raw materials. At present, food-grade glucose is the main raw material for the industrial production of erythritol (21, 22); therefore, it is particularly important to design and develop a chassis that enables efficient production of erythritol using glucose, instead of crude glycerol which contains harmful impurities.

Transporter engineering, including substrate intake and product output, is a vital research area for developing microbial cell factories (23, 24). Studies on substrate intake have mainly focused on improving the utilization of mixed sugars by microorganisms, such as by developing xylose transporters to accelerate the fermentation process of lignin or cellulose hydrolysate (24, 25). Regarding product output, emphasis is placed on eliminating product inhibition (23). Nevertheless, there are limited reports on the effective regulation of hexose transporter expression to improve bio-based chemical production in yeasts. Overexpression of the hexose transporter genes *HXT1* or *HXT7* in *Saccharomyces cerevisiae* leads to a slight enhancement in the rate of glucose consumption and production of ethanol or lactic acid (26, 27). Qiao et al. increased the ethanol productivity of *Candida glycerinogenes* recombinant strain by 25% through overexpression of hexose transporter mutant Cghxt4.2A (28). In these studies, the improvement in substrate utilization efficiency did not exceed 30%, which may be due to the oversight in collaboratively editing endogenous synthetic pathways.

In *Y. lipolytica*, transporter engineering was successfully used to produce isocitric acid and succinic acid by altering the selectivity of organic acid transport or secreting products to reduce inhibition (29, 30). A variety of glycerol transporters were used to improve the efficiency of *Y. lipolytica* by using glycerol to produce erythritol (31). The highest productivity reached 1.33 g/(L⋅h), which was still low compared with 2.84 g/(L⋅h) in other yeasts (31, 32). Previous studies have demonstrated that combined overexpression of hexokinase and hexose transporters notably hastened the production of citric acid and improved the pentose phosphate pathway, resulting in the release of polyols (33). This helps us to understand whether overexpression of the hexose transporter can further enhance the performance of the industrial erythritol-producing *Y. lipolytica*.

In this study, we reported for the first time that combining transporter and pathway engineering strategies synergistically improved the KPIs of erythritol synthesis in *Y. lipolytica*. The engineered *Y. lipolytica* Ylxs48 can produce 211.07–218.33 g/L erythritol from 310 g/L glucose within 46 h under a batch culture in 3, 100, and 200 L bioreactors as compared to 173.04–178.85 g/L for the parental strain Ylxs01 in 72 h. The productivity of erythritol reached 4.62 g/(L·h), which was 92.5% higher than that of the control strain Ylxs01 under 200 L batch fermentation. This will significantly shorten the production time and reduce the erythritol production cost. The high efficiency of the cells also allowed multiple feeding operations to improve cell utilization. After three continuous glucose feedings, the erythritol titer reached 355.81 g/L, which was the highest titer ever reported. Additionally, the erythritol production cost was reduced by 23%. Our strategy provided a new strategy for the metabolic modification of other industrial strains.

## MATERIALS AND METHODS

### Materials

All primers were synthesized by Genewiz (Suzhou, China). Super-fidelity DNA polymerase was obtained from Vazyme Biotech Co., Ltd (Nanjing, China), while all restriction endonucleases were purchased from New England Biolabs (Ipswich, MA, USA). DNA purification and plasmid purification kits were provided by Magen Co., Ltd (Shanghai, China). EasyFusion Assembly Master Mix Cloning Kit was obtained from NCM Biotech Co., Ltd (Suzhou, China). Other chemical reagents, such as defoamer, were acquired from Beijing InnoChem Science & Technology Co., Ltd (Beijing, China).

### Media and culture conditions

The *Escherichia coli* strains were cultured at 37°C and 200 rpm in Luria–Bertani (LB) medium with the addition of ampicillin (100 mg/L). The *Y. lipolytica* strains were cultivated at 30°C and 220 rpm on YPD plates containing 10 g/L yeast extract, 5 g/L tryptone, 10 g/L glucose, and 20 g/L agar. Mycophenolic acid at a concentration of 300 mg/L was included in the YNB medium (6.7 g/L yeast nitrogen base without amino acids, 3.75 g/L ammonium sulfate, and 10 g/L glucose) as needed for selecting transformants. The fermentation method for erythritol production is described in our previously published paper (34); briefly, 20 mL YPNP medium (8 g/L yeast extract, 2 g/L tryptone, 4 g/L ammonium citrate, and 3 g/L diammonium hydrogen phosphate) supplemented with glucose (310 g/L) was used and cultivated at 30°C and 220 rpm in 250 mL baffled flasks. All the cultures were weighed with flasks at the start of fermentation and before withdrawing a 200 µL sample for analysis. The reduced weight was replenished by adding the same weight of sterilized water. If necessary, polydimethylsiloxane defoamer was added to prevent the formation of foam during the fermentation.

### Strains and plasmids

Table S1 listed genes that were overexpressed in *Y. lipolytica*. Table 1 and Table S2 included detailed lists of all strains and plasmids used in this study. The *Y. lipolytica* HCY115 strain was genetically modified to enhance the efficiency of homologous recombination by deleting *KU70*, eliminate the production of byproduct mannitol by deleting the gene *MDH2*, reduce the consumption of erythritol at the end of fermentation by deleting the gene *EYD1*, and delete *ura3* as a marker (35). The *Cre* gene under the control of the *GAL1* promoter was integrated into the HCY115 genome by non-homologous recombination with *ura3* as a screening marker to obtain the *Y. lipolytica* strain Ylxs01 (34).

**TABLE 1:** Strains used in this study^*a*^

Strain	Description	Reference
*E. coli* TOP10	F– *mcr*A Δ(*mrr-hsd*RMS-*mcr*BC) Φ80 *lac*Z ΔM15 Δ*lac*X74 *rec*A1 *ara*D139 Δ(*ara- leu*) 7697 *gal*U *gal*K *rps*L (*Str^r^*) *end*A1 *nup*G	Thermo Fisher Scientific
*Y. lipolytica* HCY115	wide type (WT)CGMCC7326-YlxsWT derivative, Δ*KU70*Δ*MDH2*Δ*EYD1*Δ*ura3*	(35)
Ylxs01	HCY115 derivative, random insertion of *Cre* under P_GAL1_, using *ura3* as a marker	(34)
Ylxs02	Ylxs01 derivative, intA:: P_tef1_-*ZWF1-*T_xpr2_	This work
Ylxs03	Ylxs01 derivative, intA:: P_Php4d_-*ZWF1-*T_xpr2_	This work
Ylxs04	Ylxs01 derivative, intA:: P_hp4d_-*GND1-*T_xpr2_	This work
Ylxs05	Ylxs01 derivative, intA:: P_hp4d_-*RPE1-*T_xpr2_	This work
Ylxs06	Ylxs01 derivative, intA:: P_hp4d_-*RPI1-*T_xpr2_	This work
Ylxs07	Ylxs01 derivative, intA:: P_hp4d_-*TKL1*-T_xpr2_	This work
Ylxs08	Ylxs01 derivative, intA:: P_hp4d_-*TAL1*-T_xpr2_	This work
Ylxs09	Ylxs01 derivative, intA:: P_hp4d_-*PGI1*-T_xpr2_	This work
Ylxs10	Ylxs01 derivative, intA:: P_hp4d_-*PFK1*-T_xpr2_	This work
Ylxs11	Ylxs01 derivative, intA:: P_hp4d_-*FBA1*-T_xpr2_	This work
Ylxs12	Ylxs01 derivative, intA:: P_hp4d_-*TPI1*-T_xpr2_	This work
Ylxs13	Ylxs01 derivative, intA:: P_hp4d_-*ER10*-T_xpr2_	This work
Ylxs14	Ylxs01 derivative, intA:: P_hp4d_-*ER16*-T_xpr2_	This work
Ylxs15	Ylxs01 derivative, intA:: P_hp4d_-*ER25*-T_xpr2_	This work
Ylxs16	Ylxs01 derivative, intA:: P_hp4d_-*ER27*-T_xpr2_	This work
Ylxs17	Ylxs01 derivative, intA:: P_hp4d_-*YHT1*-T_xpr2_	This work
Ylxs18	Ylxs01 derivative, intA:: P_hp4d_-*YHT3*-T_xpr2_	This work
Ylxs19	Ylxs01 derivative, intA:: P_hp4d_-*YHT4*-T_xpr2_	This work
Ylxs20	Ylxs01 derivative, intA:: P_hp4d_-*YHT5*-T_xpr2_	This work
Ylxs21	Ylxs01 derivative, intA:: P_hp4d_-*GLK1*-T_xpr2_	This work
Ylxs22	Ylxs01 derivative, intA:: P_hp4d_-*HXK1*-T_xpr2_	This work
Ylxs23	Ylxs01 derivative, intA:: P_hp4d_-*SpHXK1*-T_xpr2_	This work
Ylxs24	Ylxs01 derivative, intA:: P_hp4d_-*AMPD*-T_xpr2_	This work
Ylxs25	Ylxs01 derivative, intA:: P_hp4d_-*DID2*-T_xpr2_	This work
Ylxs26	Ylxs01 derivative, intA:: P_hp4d_-*PYP1*-T_xpr2_	This work
Ylxs27	Ylxs01 derivative, intA:: P_hp4d_-*Vhb*-T_xpr2_	This work
Ylxs28	Ylxs01 derivative, intA:: P_3_-*ScTHI13*-T_xpr2_	This work
Ylxs29	Ylxs01 derivative, Δ*Snf1*	This work
Ylxs30	Ylxs07 derivative, *MHY1*:: P_hp4d_-*TKL1*-T_xpr2_	This work
Ylxs31	Ylxs30 derivative, *CLA4*:: P_hp4d_-*TAL1*-T_xpr2_	This work
Ylxs32	Ylxs31 derivative, intB:: P_hp4d_-*HXK1*-T_xpr2_	This work
Ylxs33	Ylxs32 derivative, intC1:: P_hp4d_-*YHT4*-T_xpr2_	This work
Ylxs34	Ylxs33 derivative, intE3:: P_hp4d_-*PYP1*-T_xpr2_, P_exp1_-*ER27*-T_lip2_	This work
Ylxs35	Ylxs33 derivative, intC2:: P_hp4d_-*GLK1*-T_xpr2_, P_exp1_-*SpHXK1*-T_lip2_	This work
Ylxs36	Ylxs33 derivative, intC3:: P_hp4d_-*YHT3*-T_xpr2_, P_exp1_-*YHT1*-T_lip2_	This work
Ylxs37	Ylxs33 derivative, intE2:: P_tef1_-*GND1*-T_cyc1_, P_hp4d_-*ER25*-T_xpr2_	This work
Ylxs38	Ylxs33 derivative, intE4:: P_hp4d_-*RPI1*-T_xpr2_, P_exp1_-*TAL1*-T_lip2_	This work
Ylxs39	Ylxs33 derivative, intF2:: P_3_-*ScTHI13*-T_xpr2_, P_hp4d_-*ER10*-T_xpr2_	This work
Ylxs40	Ylxs33 derivative, intF3:: P_hp4d_-*DID2*-T_xpr2_, P_exp1_-*Vhb*-T_lip2_	This work
Ylxs41	Ylxs34 derivative, intD1:: P_gpd1_-*PGI1*-T_xpr2_, P_hp4d_-*ER16*-T_xpr2_	This work
Ylxs42	Ylxs41 derivative, intE4:: P_hp4d_-*RPI1*-T_xpr2_, P_exp1_-*TAL1*-T_lip2_	This work
Ylxs43	Ylxs42 derivative, intF2:: P_3_-*ScTHI13*-T_xpr2_, P_hp4d_-*ER10*-T_xpr2_	This work
Ylxs44	Ylxs43 derivative, intC3:: P_hp4d_-*YHT3*-T_xpr2_, P_exp1_-*YHT1*-T_lip2_	This work
Ylxs45	Ylxs44 derivative, *ura3*:: P_tef1_-*GLK1*-T_cyc1_, P_hp4d_-*TPI1*-T_xpr2_	This work
Ylxs46	Ylxs45 derivative, intE2:: P_tef1_-*PFK1*-T_cyc1_, P_hp4d_-*FBA1*-T_xpr2_	This work
Ylxs47	Ylxs46 derivative, intF3:: P_hp4d_-*DID2*-T_xpr2_, P_exp1_-*Vhb*-T_lip2_	This work
Ylxs48	Ylxs47 derivative, intC1:: P_hp4d_-*YHT4*-T_xpr2_, P_exp1_-*YHT5*-T_lip2_	This work

^*a*^ The full names of all genes are shown in Table S1. *Sp: Schizosaccharomyces pombe; Sc: Saccharomyces cerevisiae*.

The schematic diagram of the strain construction process is shown in Fig. S1. In order to construct the single gene overexpression or deletion strains (Ylxs02–Ylxs29), homologous recombinant vectors for gene overexpression or deletion were constructed and transferred into the intA loci (36) or the target genes of the parental strain *Y. lipolytica* Ylxs01 after linearization with *Not*I. The intA and other loci were selected for gene overexpression as they displayed high gene expression levels, and the growth rate of the strains was not affected by the integration of the GFP fluorescence expression cassette (36). For multiple rounds of gene integration (Ylxs30–Ylxs48), the *guaB* marker was recovered by the *Cre-loxp* system (34). The detailed plasmid construction procedures were included in the Supporting Information (Methods), while the primers used for plasmid construction and real-time fluorescent quantitative PCR are listed in Tables S3 to S6. The process of identifying and evaluating *Y. lipolytica* transformants was described in detail in our previously published paper (35).

### Batch and fed-batch fermentation in 3, 100, and 200 L bioreactors

Batch fermentation was conducted in the 3 L bioreactor (Model BF-115, New Brunswick, USA) following the previously reported method, with a minor modification involving the adjustment of the starting glucose content in the YPNP medium from 300 to 310 g/L (34). Four different feeding strategies were implemented during the fed-batch fermentation process in 3 L bioreactors. All processes were initiated as batch cultures (1.5 L) with an initial glucose concentration of 310 g/L (total of 465 g glucose). Once the glucose in the fermentation broth was exhausted, 100 mL glucose solution was fed with one, two, or three doses of 120 or 90 g glucose, resulting in total glucose concentrations of 366 g/L (465 + 120 g), 397 g/L (465 + 120 + 90 g), 415 g/L (465 + 120 + 120 g), and 442 g/L (465 + 120 + 120+90 g) respectively. Fermentation was continued until glucose and fructose were completely exhausted.

A pilot-scale experiment was conducted in a 100 L (Model BSJ15-100, Bailun Biotech, China) or 200 L (Model R12002, Kehai Biotech, China) bioreactor containing 50 or 140 L of the YPNP medium (310 g/L glucose) and fermented at 30°C with agitation of 500 rpm. The pH did not adjust (initial pH 6.5 ± 0.2), and the aeration rate was maintained at 1 vvm (volume/culture volume/min) during the whole fermentation process. A 100 L fed-batch fermentation was conducted using an initial volume of 40 L of YPNP medium supplemented with 310 g/L glucose. More glucose (3,200, 3,200, and 2,400 g) was introduced separately to the bioreactor in triplicate at 4, 4, and 2 L, resulting in a total glucose concentration of 424 g/L (volume as 50 L). In the 200 L fed-batch fermentation, glucose in the form of dry powder was added to the bioreactor three times. The first two additions were 11.2 kg each, while the third addition was 8.4 kg, resulting in a total glucose concentration of 530 g/L (volume as 140 L). The polydimethylsiloxane defoamer was introduced to avoid foaming during fermentation when necessary.

### Analytical methods

The cell growth was monitored at a wavelength of 600 nm (OD_600_) by a UV-7504 spectrophotometer. One milliliter of cells at an OD_600_ of 1 was equivalent to 0.25 mg of dry cell weight (DCW) (35). Yeast cells were observed under optical microscopy (640×). Sugar and polyol concentration and fermentation parameters were calculated following the methodology described in our earlier study (35). Briefly, the mass yield of erythritol (Y_ERY_) was expressed in g/g from glucose and calculated from the equation Y_ERY_ = *P*/S. The volumetric productivity (Q_ERY_) was expressed in g/(L·h) and calculated from Q_ERY_ = *P*/(*V*·*t*), where *P* is the total amount of erythritol in the supernatant at the end of fermentation (g); *S* is the total amount of consumed glucose (g); *V* is the initial volume of culture liquid (L), and *t* is the fermentation time (h). The specific erythritol productivity based on the cell biomass (R_ERY_) was calculated as the amount of produced erythritol per hour and per g of DCW. RNA isolation and gene transcription levels were performed according to our previously published method (35). The citric acid was detected by a high performance liquid chromatography (HPLC) instrument (Waters Alliance e2695 HPLC, Waters, USA) with a Bio-Rad Aminex HPX-87H column. The elution condition was maintained at 50°C, 4 mM sulfuric acid, and 0.6 mL/min flow rate (37).

## RESULTS AND DISCUSSION

### Screening and characterization of overexpressed individual genes in erythritol-producing yeast *Y. lipolytica*

**Fig 1:**
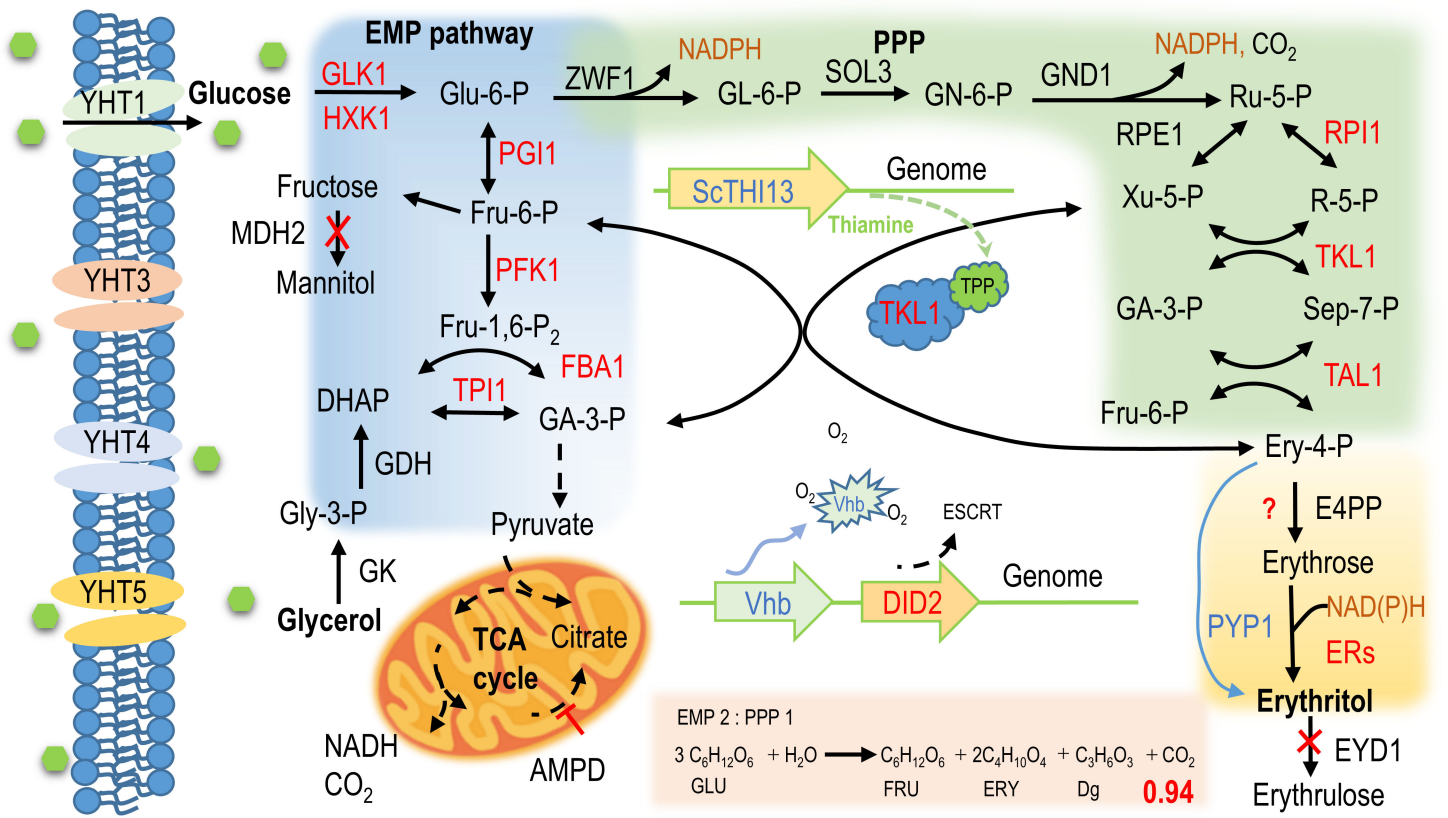
Scheme of erythritol biosynthesis in *Y. lipolytica*. Endogenous enzymes or pathways are indicated in black and red (endogenous genes are overexpressed). Heterologous enzymes or pathways are indicated in blue. Dashed arrows represent multiple steps. PPP, pentose phosphate pathway; Glu-6-P, glucose-6-phosphate; GL-6-P, 6-phosphogluconolactone; GN-6-P, 6-phosphogluconate; Ru-5-P, ribulose-5-phosphate; R-5-P, ribose-5-phosphate; Xu-5-P, xylulose-5-phosphate; Sep-7-P, sedo-heptulose-7-phosphate; EMP pathway, Embden–Meyerhof–Parnas pathway; Fru-6-P, fructose-6-phosphate; Fru-1,6-P_2_, fructose-1,6-bisphosphate; DHAP, dihydroxyacetone phosphate; Gly-3-P, glycerol-3-phosphate; GA-3-P, glyceraldehyde-3-phosphate; Ery-4-P, erythrose-4-phosphate; GLU, glucose; FRU, fructose; ERY, erythritol; Dg, glyceraldehyde; TCA cycle, tricarboxylic acid cycle; full name of all genes are shown in Table S1. The theoretical highest yield is that the carbon metabolic flow is distributed in the form of EMP: PPP 2:1, and the final yield of erythritol is 0.94 g/g glucose. The calculation of the above erythritol yield assumes that only CO_2_ is calculated as carbon loss and that the other products produced can be reused. Other biological processes, such as biomass synthesis, were not considered.

The genes involved in erythritol biosynthesis and catabolism have been functionally characterized (38, 39). They are mainly derived from the Embden–Meyerhof–Parnas (EMP) pathway and pentose phosphate pathway (PPP). Key intermediate metabolites include fructose-6P (Fru-6P), glyceraldehyde-3-phosphate (GA-3P), xylulose-5-phosphate (Xu-5P), and erythrose-4-phosphate (Ery-4P). This latter molecule (Ery-4P) was subsequently converted into erythrose by erythrose-4P-phosphatase, the gene responsible for which remained to be discovered in *Y. lipolytica*. The final step for erythritol synthesis involves the reduction of erythrose by erythrose reductase (ER) in the presence of NAD(*P*)H (Fig. 1). So far, a total of eight ER homologous genes have been characterized in *Y. lipolytica*. However, their deletion did not eliminate the production of erythritol, suggesting the existence of undiscovered ER or ER-like enzymes (40). Overexpression of genes involved in those pathways, such as transketolase (*TKL1*) or erythrose reductase (*ER*), led to a significant increase in erythritol titer and productivity (41, 42). As erythritol synthesis was not a redox-balanced reaction, the expression of genes leading to the formation of NADPH, namely *ZWF1* and *GND1*, encoding glucose-6-phosphate dehydrogenase and 6-phosphogluconate dehydrogenase, respectively, had a positive impact on erythritol synthesis (35).

Various strain engineering strategies to increase the flux in carbon source catabolism to increase the intracellular levels of Ery-4P also succeeded in increasing both P_ERY_ (production or titer, g/L) and Q_ERY_ [productivity, g/(L·h)]. For instance, overexpression of glycerol catabolic genes, namely *GUT1* and *GUT2* encoding glycerol kinase and glycerol-3P dehydrogenase, respectively, resulted in a substantial increase in the rate of glycerol uptake and erythritol productivity (15, 42). Noteworthy, these two studies showed that the strain’s genetic background was an important factor as it can modulate the gene overexpression on both P_ERY_ and Q_ERY_. In *Y. lipolytica* strain A101, the overexpression of *GUT1* and *GUT2* resulted in a 35% increase in Q_ERY_ (15), while in a derivative of strain W29, the overexpression of both genes led to a decrease in Q_ERY_ compared with the overexpression of *GUT1* alone (42).

Overexpression of genes that were not directly associated with erythritol synthesis has also been demonstrated to be beneficial for erythritol production. For instance, the expression of codon-optimized bacterial hemoglobin from *Vitreoscilla stercoraria* (Vhb) resulted in an 83% higher P_ERY_ (43). Recently, the functionality of six hexose transporters, four of which had the ability to transport glucose (i.e., *YHT1*, *YHT3*, *YHT4*, and *YHT5*), as well as hexokinase (*GLK1*, *HXK1*, and heterologous *SpHXK1*), had been investigated in *Y. lipolytica* (44, 45). Overexpressing some of these genes in *Y. lipolytica* increased the carbon flux in the PPP, thus improving the production of polyols (33).

**Fig 2:**
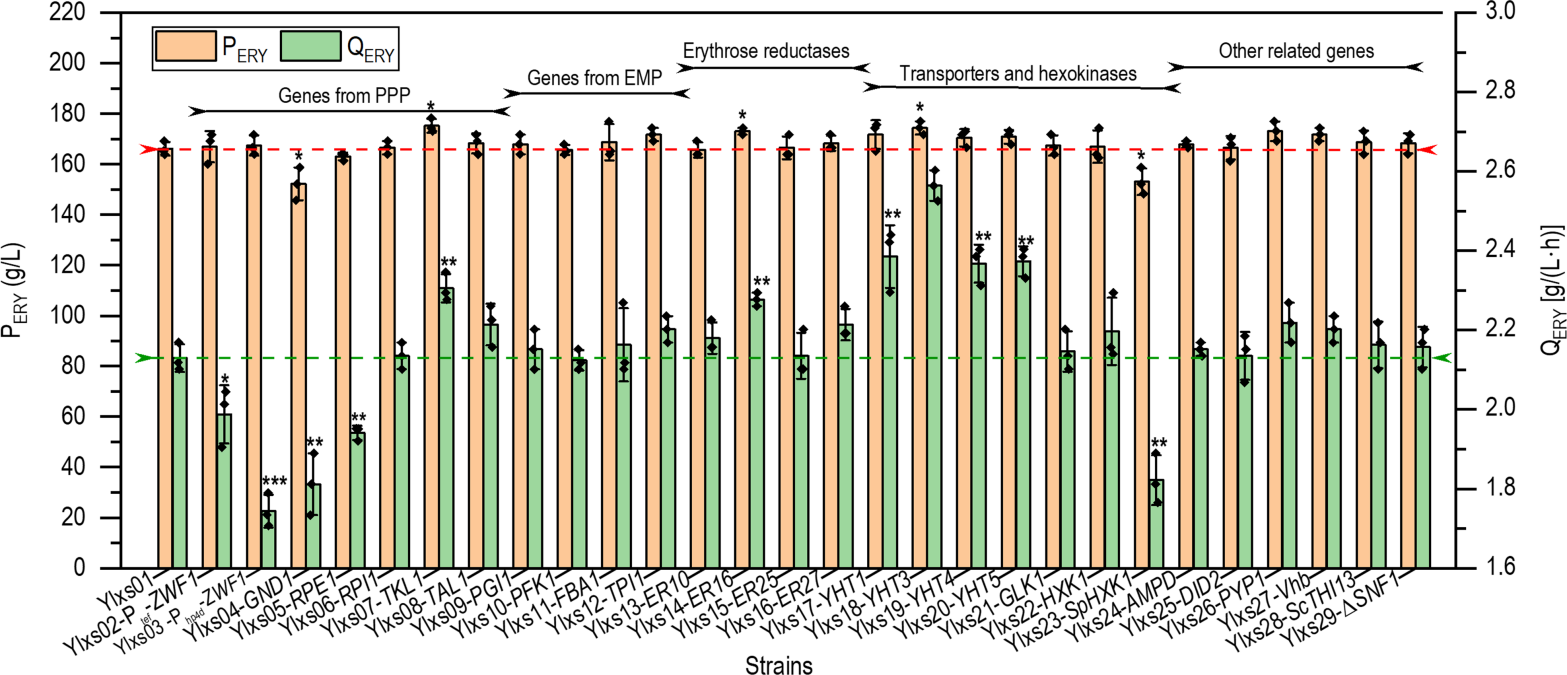
Single gene screening and characterization of the engineered *Y. lipolytica*. All strains were cultured in 250 mL baffled flasks containing 20 mL of YPNP medium supplemented with 310 g/L glucose. The fermentation ended at different time points when the carbon source was completely consumed. Error bars represent standard deviation (*n* = 3). One-way analysis of variance (ANOVA) was applied to check the significance of the data (*P < 0.05, **P < 0.01, ***P < 0.001, ****P < 0.0001, and NS not significant). NS was not shown in the figures.

Genes associated with the pentose phosphate (PP) and Entner–Doudoroff pathways (EMP), erythrose reduction (ER), glucose transport, and other related functions (not directly linked to erythritol synthesis or glucose utilization) were also examined. They were cloned under the control of the strong constitutive promoter P_hp4d_ and integrated individually at the intA locus of *Y. lipolytica* strain Ylxs01, a wild-type strain CGMCC7326 derivative previously obtained by the disruption of genes *MDH2* and *EYD1* gene encoding mannitol and erythritol dehydrogenase, respectively (34). After confirming the corresponding gene overexpression by qPCR, the resulting strains, namely Ylxs03 to Ylxs27, were grown in YPNP medium containing glucose (310 g/L). The calculated erythritol titer (P_ERY_, g/L) and productivity [Q_ERY_, g/(L·h)] were displayed in Fig. 2.

Among the genes involved in PPP, only the overexpression of the gene *TKL1* encoding transketolase showed an increase in both P_ERY_ and Q_ERY_ (5% and 8%, respectively; strain Ylxs07) compared with the parental strain Ylxs01 [166.11 g/L, 2.13 g/(L·h), respectively], reaching 175.24 g/L and 2.31 g/(L·h), respectively. In the strain Ylsx08 (overexpression of transaldolase, *TAL1*), Q_ERY_ was slightly increased (4%) but not P_ERY_, while in strain Ylsx06, overexpression of *RPI1* (ribulose-5P isomerase) did not affect P_ERY_ and Q_ERY_. In contrast, overexpression of *ZWF1* (glucose-6P dehydrogenase, strain Ylxs03), *GND1* (6-phosphogluconate dehydrogenase, strain Ylxs04), and *RPE1* (ribulose-5P epimerase, strain Ylxs05) led to slightly lower Q_ERY_. In the literature, the results obtained from *ZWF1* and *GND1* overexpression were contradictory (41, 46–48). Although our findings aligned with the data obtained from *Y. lipolytica* strain MK1 (46), the results obtained from strain PO1f indicated enhanced P_ERY_ for both genes overexpression (47, 48). The results validated that the different genetic backgrounds or metabolisms of specific strains might influence the effect of specific gene expression on erythritol synthesis.

Considering the genes involved in the EMP pathway, their overexpression did not lead to a significant increase in P_ERY_ and Q_ERY_. Overexpression of ER genes resulted in increased P_ERY_ and Q_ERY_ values for gene *ER16*, with a slight increase of 4% and 7%. The overexpression of *ER10*, *ER25*, and *ER27* did not have an obvious impact on erythritol P_ERY_ and Q_ERY_. Overexpression of hexose transporter *YHT3* (strain Ylxs18) led to a significant increase in Q_ERY_ [20%; 2.56 g/(L·h)] and a slight increase in P_ERY_ (5%) as compared to the parental strain Ylxs01. When genes *YHT1*, *YHT4*, and *YHT5* were overexpressed, there was a notable rise in Q_ERY_ (12%, 11%, and 11% correspondingly). However, the corresponding P_ERY_ values showed no significant change compared with the control strain Ylxs01. In contrast, the overexpression of hexokinase or glucokinase had no beneficial effect. This illustrated that glucose transportation is one of the limiting steps for high erythritol productivity.

For the other related genes, none of them (*PYP1*, *AMPD*, *DID2*, *ScTHI13*, *Vhb*, and *SNF1*) achieved a significant increase (<4%) in erythritol production, although previous studies showed that heterologous overexpression of the sugar alcohol phosphatase (PYP1) (49), bacterial hemoglobin Vhb (43), and AMP deaminase (AMPD) (47) increased erythritol production in *Y. lipolytica*. Previous studies also showed that overexpression of the *DID2* gene, a subunit of the endosomal sorting complex required for transport (ESCRT), increased PPP expression (*ZWF1* and *TKL1*) and promoted glucose utilization (50). However, we did not observe accelerated consumption of glucose in the erythritol-producing *Y. lipolytica* Ylxs25 (intA:: P_hp4d_-*DID2*-T_xpr2_). The disruption of sucrose non-fermenting (*SNF1*) protein kinase in *Y. lipolytica* enhanced erythritol production under nitrogen-enriched fermentation conditions (51). We deleted the *SNF1* of the strain Ylxs01, and no significant change was observed with the erythritol production of strain Ylxs29 (Δ*SNF1*) under our culture conditions (Fig. 2). Since thiamine activates transketolase (TKL1), a key enzyme in erythritol biosynthesis, an engineered *Y. lipolytica* strain (Ylxs28) was therefore constructed by integrating the heterologous *THI13* gene from *S. cerevisiae* under the thiamine-regulated promoter P3 (52, 53). The erythritol production of the resulting strain Ylxs28 (intA:: P_3_-*ScTHI13*-T_xpr2_) did not change significantly. Although these genes did not play a significant role in enhancing erythritol production when overexpressed individually, there may be potential beneficial effects when combined expressions were conducted, resulting in synergistically accumulated effects.

### Combined hexose transport and pathway engineering to improve the erythritol titer and productivity in *Y. lipolytica*

The overexpression of individual genes from the above-mentioned five groups yielded an increased erythritol production titer and productivity with varying degrees of success. It has been reported that a significant increase in erythritol titer, productivity, and yield can be achieved by expressing multiple genes aiming to augment carbon flux into the EMP and redirect it through PPP. For example, the pull-and-push strategy used by Carly et al. (42), which aimed to redirect the carbon flux from glycerol to erythritol, resulted in a significant increase (42% and 75%, respectively) in production titer and productivity (42). However, these studies mainly focused on the reinforcement of endogenous pathways. Unlike glycerol, glucose must be assisted by transporters to enter cells, and substrate transport was proved to be a major obstacle to efficient erythritol production from glucose (Fig. 2). Therefore, we first applied a similar strategy to redirect the carbon flux from glucose to erythritol, and then combined it with the enhancement of glucose transport. The strain Ylxs07, which overexpressed *TKL1*, was used as the starting strain because the C-flux from the EMP is redirected to the PPP (46).

**Fig 3:**
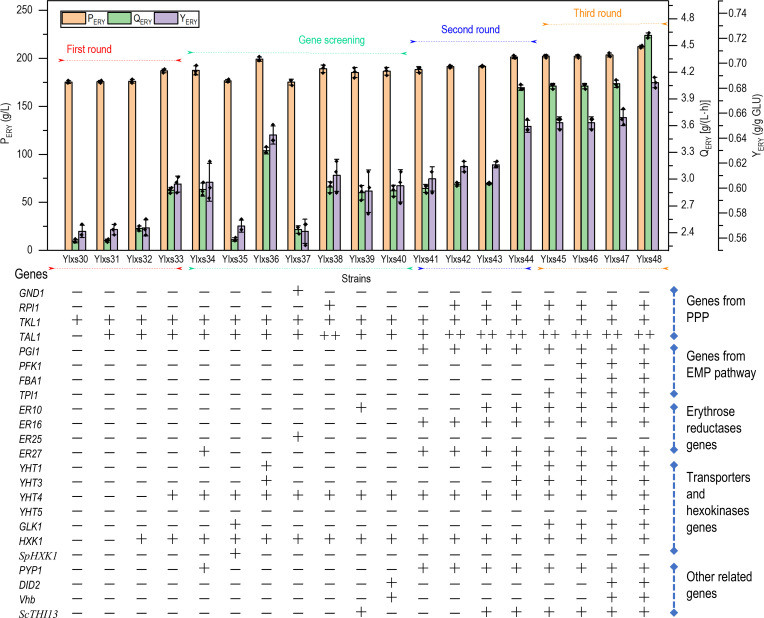
Effects of combination expression of transporter engineering and pathway engineering on erythritol synthesis performance. The engineered *Y. lipolytica* strains were incubated for erythritol production in 250 mL baffled flasks containing 20 mL of YPNP medium supplemented with 310 g/L glucose. The strain Ylxs07 (Ylxs01 derivative, intA:: P_hp4d_-*TKL1*-T_xpr2_) was used as the parental strain to be further modified. The “+” represents one round of genetic manipulation. Error bars represent standard deviation (*n* = 3).

The genes *TKL1*, *TAL1*, *HXK1,* and *YHT4* were first sequentially overexpressed in strain Ylxs07, resulting in strains Ylxs30 to Ylxs33. The *TAL1* and the additional copy of *TKL1* genes were integrated at *CLA4* and *MHY1* locus, respectively, because the knockout of *MHY1* or/and *CLA4* had no significant impact on the stress tolerance of *Y. lipolytica* (34), leading to non-filamentous strains (Fig. S2), which are of technological advantage for cell growth in bioreactors. Overexpression of *TAL1* or an additional copy of *TKL1* did not significantly increase the erythritol titer and productivity for the corresponding strains (Fig. 3). When evaluating HXK1, the only notable difference was a minor increase (6%) in the productivity compared to the parental strain Ylxs07. In contrast, when *YHT4* was overexpressed in strain Ylxs33, the productivity and yield were increased remarkably (35% and 11%, respectively), in comparison to the parental strain Ylxs01. Erythritol titer for the strain Ylxs33 reached 186.98 g/L, which is 12% higher than that of the parental strain Ylxs01.

To further improve erythritol production, various genes from the four above-mentioned groups were overexpressed in strain Ylxs33, generating strain Ylxs34–Ylxs40. As shown in Fig. 3, the co-overexpression of *SpHXK1* (Ylxs35) or *GND1* (Ylxs37) had a notable negative impact on erythritol titer, which aligned with the findings from the individual gene screening (Fig. 2). The erythritol titer, productivity, and yield of the strain Ylxs36 overexpressing *YHT1* and *YHT3* were significantly improved to 199.15 g/L, 3.32 g/(L·h), and 0.64 g/g glucose, which also demonstrated the importance of improving sugar transport. As a result, two additional rounds of integration were performed (in the second round, Ylxs34 was used as the parental strain, resulting in the strains from Ylxs41 to Ylxs44; in the third round, Ylxs44 was used as the parental strain, resulting in the strains from Ylxs45 to Ylxs48). As shown in Fig. 3, although the overexpression of the endogenous pathway did not significantly increase the KPI parameters (P_ERY_, Q_ERY_, and Y_ERY_) of erythritol synthesis, it had a significant superposition effect when synergistically expressed with the transporter pathway genes.

After multiple integrations of genes involved in the glucose transporter pathway and erythritol endogenous synthetic pathway, the final engineered strain Ylxs48 was obtained (The detailed strain construction process was shown in Fig. S1). The titer, productivity, and yield of erythritol synthesis by the strain Ylxs48 were 212 g/L, 4.61 g/(L·h), and 0.68 g/g glucose, which were 27.74%, 116.43%, and 25.92% higher respectively than that of control strain Ylxs01 in 250 mL baffled flasks, especially with doubled enhancement of productivity. The total fermentation time was shortened from 78 to 46 h, which was 32 h less than that of the starting strain. (Table S8).

**Fig 4:**
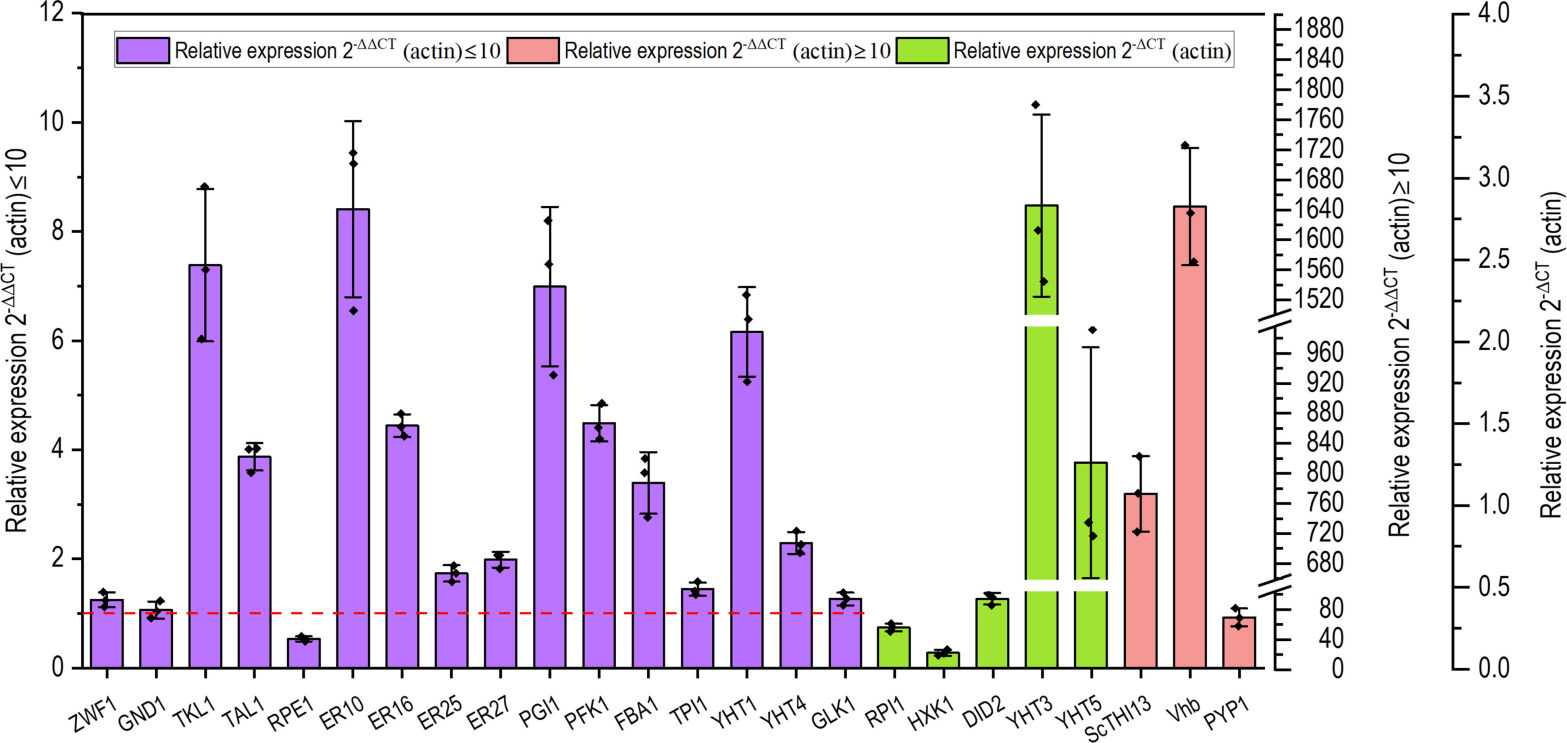
Expression profiles for genes by the engineered *Y. lipolytica* Ylxs48. The strain Ylxs48 was cultured in 250 mL baffled flasks containing 20 mL of YPNP medium supplemented with 310 g/L glucose. Gene expression levels were normalized based on the expression of the actin gene YALI0_D08272g. Error bars represent standard deviation (*n* = 3).

To better understand the mechanism of increased efficiency of erythritol production for the engineered *Y. lipolytica* Ylxs48, we measured the expression of the genes involved in glucose uptake and erythritol synthesis using quantitative PCR. As shown in Fig. 4, the expression levels of all overexpressed genes were upregulated to varying degrees compared with the control strain Ylxs01. Among them, the gene *YHT3* encoding the glucose transporter was significantly upregulated, which partly accounted for the remarkable increase in glucose consumption for the strain Ylxs18 when *YHT3* was overexpressed (Fig. 2).

The expression of key genes *TKL1*, *TAL1*, *ER10*, and *ER16* was increased 3- to 9-fold. The expression levels of *ZWF1*, *GND1,* and *ER25*, which were not overexpressed, did not significantly change. The only decreased expression was *RPE1*, possibly due to the overexpression of *PGI1*, *PFK1*, and *FBA1* in the EMP pathway and *TKL1* in PPP, which might increase the amount of xylulose-5-phosphate (Xu-5-P) and erythrose-4-phosphate (Ery-4-P) from fructose-6-phosphate (Fru-6-P) and glyceraldehyde-3-phosphate (GA-3-P) produced by the enhanced EMP pathway. Consequently, the metabolic flux from ribulose-5-phosphate (Ru-5-P) to Xu-5-P decreased. The increase in key gene expression of hexose transport and endogenous synthetic pathways enhanced glucose uptake, which provided sufficient glucose to accelerate erythritol synthesis, and then increased the titer, yield, and productivity of erythritol.

### Erythritol production in 3 L bioreactor

The engineered strain Ylxs48 and its parental strain Ylxs01 were first grown in batch mode in YPNP medium (containing 310 g/L glucose). The titer, productivity, and yield of erythritol for Ylxs01 were 177.35 g/L, 2.43 g/(L·h), and 0.57 g/g glucose, respectively, and maximal erythritol titer was obtained after 72 h (Table 2). Those results were consistent with our previous findings (34). For batch fermentation of the strain Ylxs48, the highest titer was obtained after 46 h, which represents a 36% reduction in total fermentation time (46 h versus 72 h). Moreover, the titer, productivity, and yield for Ylxs48 reached 218.33 g/L, 4.65 g/(L·h), and 0.70 g/g glucose, remarkably increased by 23%, 91%, and 23%, respectively, as compared with the control Ylxs01. The glucose consumption rate was also remarkably increased for strain Ylxs48, reaching 6.59 g/(L·h), which is 55% higher than that of the parental strain Ylxs01 4.24 g/(L·h)]. The HPLC profiles of the time course of the fermentation in 3 L bioreactors with the control strain Ylxs01 and the engineered *Y. lipolytica* Ylxs48 were shown in Fig. S3. It clearly showed that the glucose consumption rate of the engineered strain Ylxs48 was faster than that of the control strain Ylxs01.

**TABLE 2:** Key performance indicators of erythritol synthesis by engineered strain YLxs48 under batch and glucose fed-batch conditions

Strain	Glucose pulses^*a*^	Glucose added (g)	Time of addition (h)	P_ERY_ (g/L)	Y_ERY_ (g/g GLU)	Q_ERY_ [g/(L⋅h)]	T (h)	Q_GLU_ [g/(L⋅h)]	R_ERY_ [g/(g DCW·h)]
Ylxs01	0	0	-^*b*^	177.35	0.57	2.43	72	4.24	0.24
Ylxs48	0	0	-	218.33	0.70	4.65	46	6.59	0.48
Ylxs48	1	120	46	273.99	0.74	4.64	58	6.13	0.50
Ylxs48	2	120 + 90	46, 54	292.06	0.73	4.23	68	5.74	0.49
Ylxs48	2	120 + 120	46, 54	306.01	0.73	4.19	72	5.68	0.48
Ylxs48	3	120 + 120 + 90	46, 54, 63	324.53	0.72	3.82	84	5.19	0.46

^*a*^ Represents the number of glucose pulses; P_ERY_: erythritol production titer; Q_ERY_: erythritol volumetric productivity; Y_ERY_: erythritol yield; T: fermentation time when glucose was completely consumed; Q_GLU_: glucose consumption rate; R_ERY_: specific erythritol productivity based on the cell biomass; cultures were performed in 3 L bioreactor containing initial 1.5 L of YPNP medium with 310 g/L glucose; The values are the means of three independent replicates. The standard deviation represented less than 2% of the means.

^*b*^ -, no addition of glucose at this time point.

Owing to the rapid depletion of glucose, the yeast cells still maintained high viability. The viable cell count was monitored throughout the erythritol production by alkaline methylene blue staining (35). As depicted in Fig. S4A and B, at 46 h (the engineered strain Ylxs48 depleted out glucose), almost all the control and engineered strains survived, and the proportion of cells with loss of viability was less than 1.52%. At 78 h (the control strain Ylxs01 depleted out glucose), the percentage of cells loss of viability for the parental and engineered strain increased by 11.67% and 5.62% individually. Interestingly, at this time point (78 h), the cells of the engineered strain Ylxs48 remained in a circular and dispersed state (single cells), while the less active cells of the control Ylxs01 exhibited an obvious flocculation effect (aggregation in clusters), which promoted foam formation and accelerated cell sedimentation (Fig. S4C). Considering the strong viability of the engineered strain Ylxs48 at 78 h, glucose fed-batch was performed during the fermentation process at different times.

The fed-batch culture mode is a well-established strategy for increasing process titer and productivity. Pulsed-fed batch cultures were proposed using the yeast *Y. lipolytica* S3 (performed in two variants in which glycerol was dosed in four portions of about 50 or 75 g/L) resulting in increased polyol production of up to 140 or 176.4 g/L (54). With pulsed glucose feeding to a total concentration of 400 g/L (glucose dosing variant of 150 + 4 × 62.5 g/L) in the medium containing yeast extract, 208 g/L erythritol was obtained in 240 h using the yeast *Y. lipolytica* K1UV15 (55). The high yield of erythritol requires high osmotic pressure, which was achieved by a high concentration of glucose (56). However, achieving this in traditional continuous feeding culture using glucose as the carbon source is challenging. The carbon source of the feed must have a glucose content greater than 80%. Glucose at high concentration is very easy to crystallize, needs to be used immediately, and is not suitable for continuous fed-batch fermentation operation. If the glucose concentration in the feed is lower, it will result in an increased final volume of fermentation, leading to higher costs for product purification. Pulsed-fed batch culture can achieve higher glucose concentration feed and avoid crystallization of glucose in the pipeline. Therefore, pulsed-fed batch cultures were performed here. Fermentation was started in batch mode in YPNP medium (containing glucose 310 g/L) for 46 h, and upon glucose exhaustion in the culture medium, feeding was conducted stepwise in various modes by the addition of either 120 or 90 g glucose (Table 2). The erythritol titer increased by 48% after three glucose pulses from 218.33 g/L in batch mode to 324.53 g/L. Erythritol yield (0.72–0.74 g/g glucose) and R_ERY_ [0.46–0.50 g/(g DCW·h)] remained similar for the different pulse strategies tested.

**Fig 5:**
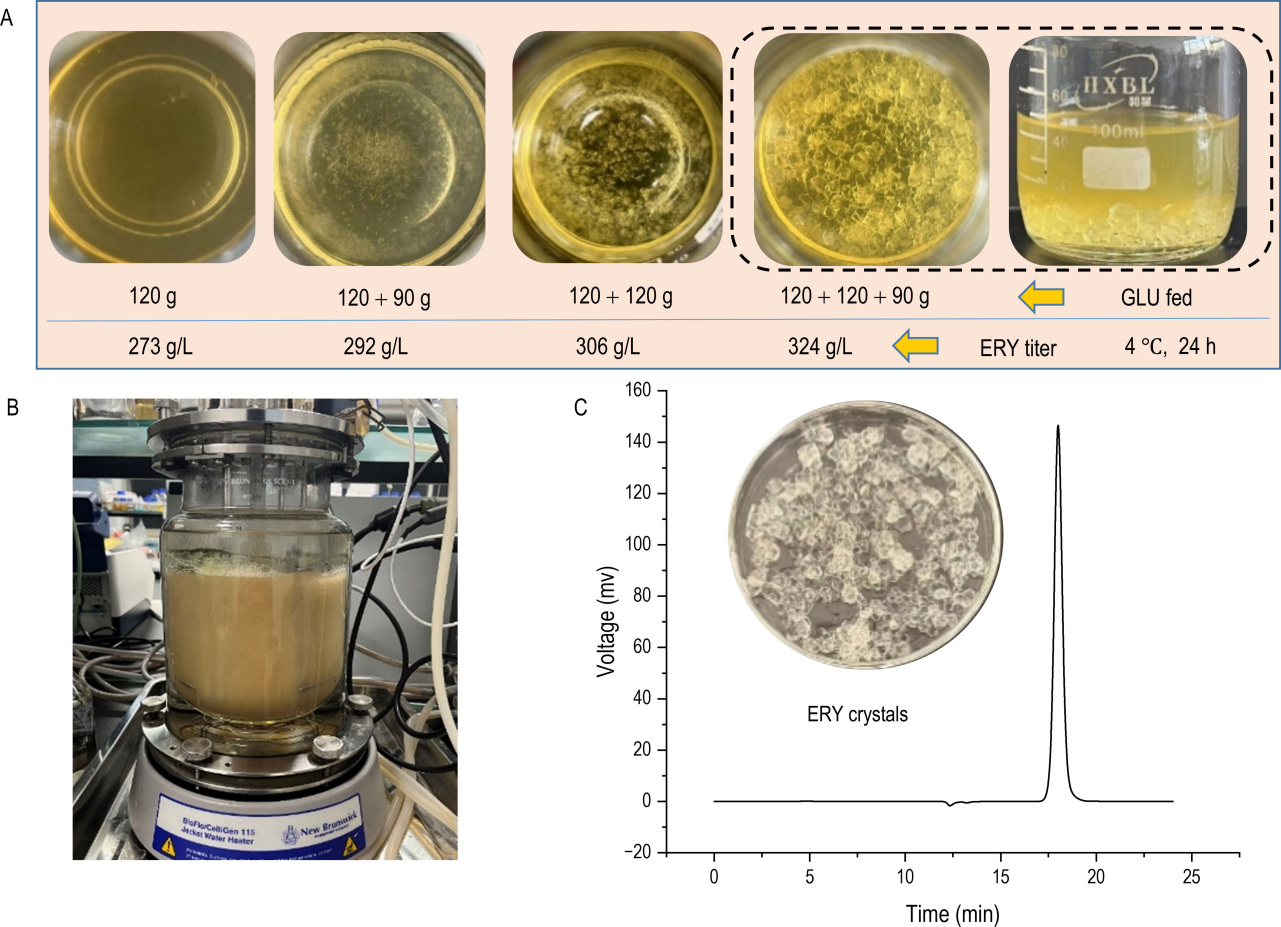
Schematic diagram of the glucose-fed fermentation process of the engineered strain Ylxs48 in the 3 L bioreactor. (A) The direct erythritol crystallization from clear fermentation supernatant at 4°C under different feeding strategies without concentration. (B) The image of erythritol production under fed-batch fermentation with the engineered strain Ylxs48 in the 3 L bioreactor. (C) The image of erythritol crystals and detection of purity by HPLC.

Direct erythritol crystallization in culture supernatant at 4°C under different feeding strategies was assessed. As shown in Fig. 5A, erythritol crystals did not appear in the supernatant obtained after one glucose feeding (120 g glucose was fed). After two successive glucose feedings (120 + 90 g glucose), the erythritol titer reached 292 g/L, and erythritol crystals formed at 4°C in the supernatant, which means that less steam was consumed for fermentation broth concentration during erythritol crystallization. The purity of the crystal was greater than 99.95%, and no other polyol impurities were present (Fig. 5C).

### Scale-up of the erythritol production in 100 and 200 L bioreactor

To assess the process scalability and characterize the production stability of Ylxs48, erythritol production was carried out at 100 and 200 L bioreactors. The parental strain Ylxs01 was first evaluated in batch. As shown in Table S9 and Table 3, the erythritol titer, productivity, and yield from glucose were 173.04–178.85 g/L, 2.4–2.63 g/(L⋅h), and 0.55–0.57 g/g glucose, respectively. As compared to the 3 L bioreactor (0.57 g/g glucose), the yield of erythritol decreased slightly under the 200 L bioreactor. The engineered *Y. lipolytica* Ylxs48 consumed 310 g/L of glucose in 43–46 h and produced 211.07–212.65 g/L erythritol, almost 32–40 g/L higher than that of the control (18%–22% higher), with a yield of 0.69 g/g. Fermentation time was reduced by 25–26 h, as compared to above 68–72 h for the parental strain Ylxs01, with productivity of 4.62–4.91 g/(L⋅h). Compared with the batch fermentation in 3 L bioreactor, there were no significant differences in the KPIs of erythritol for strain Ylxs48 (titer: 218.33 g/L; yield: 0.70 g/g; productivity: 4.65 g/[L⋅h]), indicating it was stable or reproducible in erythritol KPIs from 3 L to 100 and 200 L.

**TABLE 3:** Comparison of the reported erythritol production KPI for different yeasts

Strain	Culture system	Carbon source	Volume (L)	P_ERY_ (g/L)	Q_ERY_ [g/(L⋅h)]	Y_ERY_ (g/g)	Byproduct^*a*^	Reference
*Aureobasidium* sp. SN-G42	Batch	Glucose	3	175	1.82	0.44	NR	(4)
*Trichosporon* sp.	Fed-batch	Glucose	5	149	1.86	0.45	Glycerol	(57)
*Torula* sp.	Batch	Sucrose	5	200	1.67	0.50	NR	(58)
*Candida magnoliae* M2	Fed-batch	Glucose	50	200	1.2	0.43	NR	(59)
*Pseudozyma tsukubaensis*	Fed-batch	Glucose	50,000	241	2.84	0.6	NR	(32)
*Moniliella sp*. N61188-12	Fed-batch	Glucose	2,000	189.4	0.76	0.44	Ribitol glycerol	(60)
*Y. lipolytica* CICC 1675	Fed-batch	Glycerol	7	194.3	0.95	0.49	Mannitol	(56)
*Y. lipolytica* MK1	Repeated batch	Glycerol	5	224	0.7	0.67	Mannitol arabinitol	(12)
*Y. lipolytica* M53	Fed-batch	Glycerol	5	164.1	1.05	0.65	Mannitol arabinitol	(61)
*Y. lipolytica* yliUA8	Fed-batch	Glucose	3	148	1.56	0.59	NR	(13)
*Y. lipolytica* HCY118	Batch	Glucose	30,000	196	2.51	0.65	NO	(35)
*Y. lipolytica* Y-11	Batch	Glycerol	5	150	1.25	0.62	Mannitol arabinitol	(62)
*Y. lipolytica* E326	Fed-batch	Glucose	5	256	1.78	0.58	Mannitol arabinitol	(48)
*Y. lipolytica* Ylxs01	Batch	Glucose	200	173.04	2.40	0.55	NO	This study
*Y. lipolytica* Ylxs48	Batch	Glucose	200	212.65	4.62	0.69	NO	This study
*Y. lipolytica* Ylxs48	Fed-batch	Glucose	200	355.81	4.29	0.71	NO	This study

^*a*^ NR, not reported; NO, no polyol by-products. The high-performance (titer >140 g/L and yield >0.4 g/g) strains were selected to represent the possibility of industrial application. Only the highest erythritol titer was listed here for the same parental strain.

Previous studies have shown that overexpression of *TKL1* enabled the efficient production of erythritol regardless of the presence of dissolved oxygen (46). The engineered strain Ylxs48 also exhibited a more stable production phenotype compared with the control strain Ylxs01 during pilot-scale fermentation carried out under batch conditions. Figure S5 depicted in more detail the glucose batch fermentation process of the parental strain Ylxs01 and the engineered strain Ylxs48 in the 100 L bioreactor. The Incyte sensor (Hamilton Company) was used to monitor the viable cell density online (63). As shown in Fig. S5A and B, the viable cell density of engineered strain Ylxs48 reached a maximum of 8.02 pF/cm at 14 h, and that of parental strain Ylxs01 reached a maximum of 9.39 pF/cm 6 h later. Subsequently, the viable cell density of parental strain Ylxs01 gradually decreased to 6.4 pF/cm, while the engineered strain Ylxs48 remained stable until the end of fermentation and only slightly decreased to 7.22 pF/cm. This was consistent with what we observed in shaker culture (Fig. S4), where parental strain Ylxs01 had a higher proportion of cells loss of viability at the end of fermentation (11.67% vs 5.62%). Total biomass increased rapidly to OD_600_ 34 within 24 h, accompanied by a rapid decline in dissolved oxygen (DO) and pH. Paralle l to cell growth, oxygen demand of the cells increased during growth, showing a maximum oxygen uptake rate (OUR) of 43.72 and 53.46 mmol/(L·h), respectively, in the parental strain Ylxs01 and the engineered strain Ylxs48. Maximum carbon dioxide evolution rate (CER) at the same time was at 50.74 and 69.71 mmol/(L·h), and the average RQ during the production phase was 1.16 and 1.33 (Fig. S5C and D). Subsequently, the parental strain Ylxs01 continued to grow slowly, and the final biomass reached OD_600_ 41.28 (Fig. S5E), and the pH also slowly decreased to 2.76. After 24 h, the biomass of engineered strain Ylxs48 increased slightly to 37.12 (Fig. S5F), the DO gradually increased from 4.08% to 31.75%, and the pH stabilized at 3.03 until the fermentation ended (glucose and fructose were exhausted). The pH change was attributed to the secretion of citric acid (CA), which was 8.35 and 4.83 g /L, respectively, in the parental strain Ylxs01 and the engineered strain Ylxs48 at the end of fermentation (Table S9). As a fraction of the glucose is used for biomass and CA formation, erythritol synthesis occurred with a yield of 0.34 and 0.42 g/g glucose from 0 to 24 h of the parental strain Ylxs01 and the engineered strain Ylxs48, respectively.

The rate of glucose consumption gradually increased, reaching a maximum value of 6.39 and 11.57 g/(L·h), while the yield of erythritol reached 0.69 g/g glucose (between 36 and 48 h) and 0.78 g/g glucose (between 36 and 43 h), respectively (Fig. S5G and H). Combined with the off-gas analysis and HPLC data, we estimated the distribution of glucose consumption in the whole fermentation process. As shown in Table S10, the engineered strain Ylxs48 completed fermentation 25 h faster than the parental strain Ylxs01, thus reducing the total aerobic respiratory consumption (1,168.65 g). However, the enhancement of the pentose phosphate pathway (PPP) in the engineered strain Ylxs48 resulted in an increase in glucose loss by 117.77 g (each molecule of glucose through PPP will lose one molecule of carbon dioxide), and the total glucose loss in the form of carbon dioxide of the parental strain Ylxs01 and engineered strain Ylxs48 was 4,877.3 and 3,826.42 g, accounting for 31.46% and 24.68% of the total substrate content, respectively. The glucose loss of the engineered strain due to biomass and citric acid production was also lower than that of the parental strain, and the final calculation results were consistent with the actual conversion rate.

**Fig 6:**
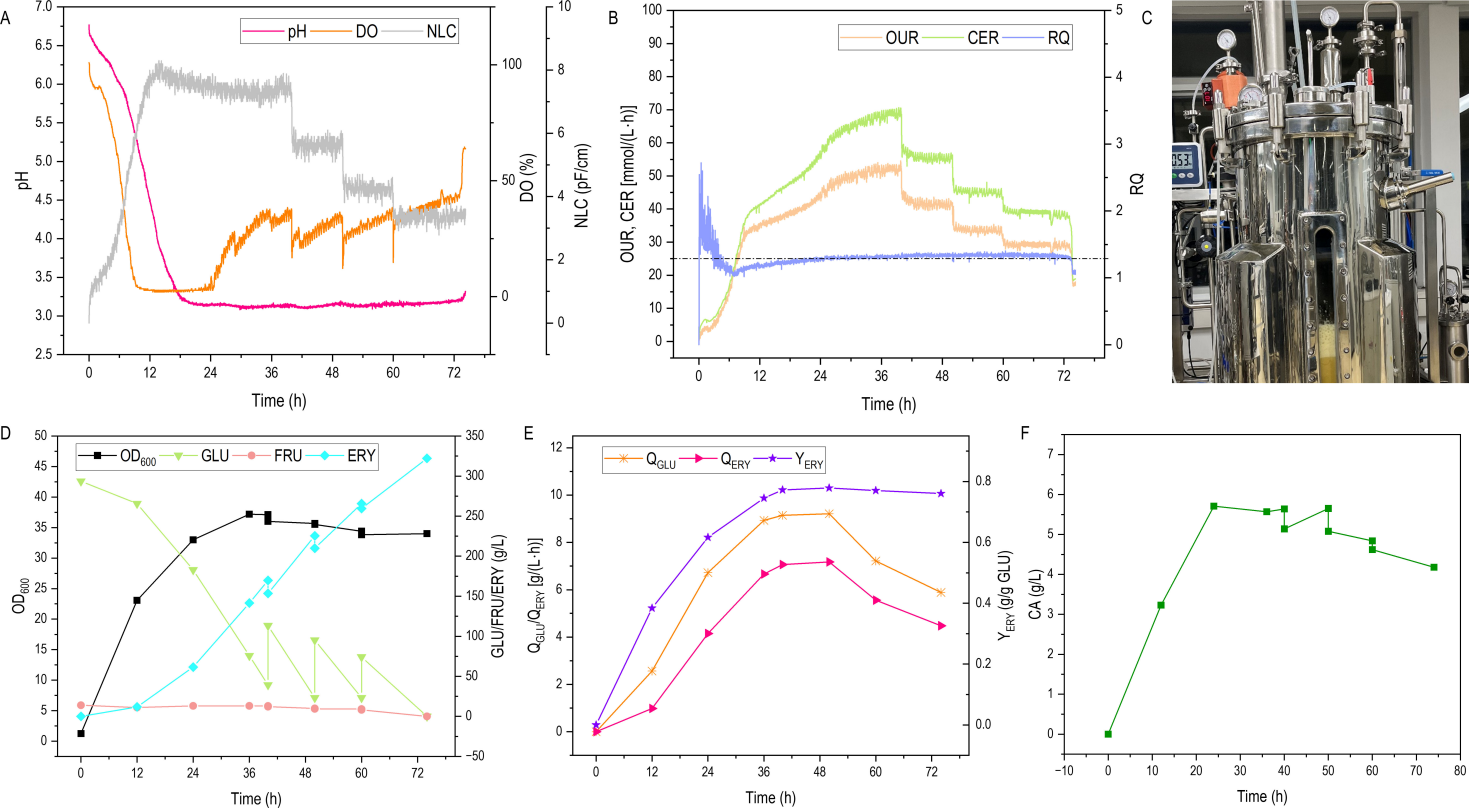
Schematic diagram of the glucose-fed fermentation process of the engineered strain Ylxs48 in the 100 L bioreactor. (A) Time course effects on cell growth (number of living cells, NLC), DO, and pH changes. (B) Time course effects on oxygen uptake rate (OUR), carbon dioxide evolution rate (CER), and respiration quotient (RQ) changes. (C) The image of erythritol production under fed-batch fermentation with the engineered strain Ylxs48 in the 100 L bioreactor. (D) Time course effects on cell growth (OD_600_), glucose, fructose, and erythritol titer changes. (E) Time course effects on glucose consumption rate (R_GLU_), productivity (Q_ERY_), and Yield (Y_ERY_) changes. (F) Time course effects on citric acid (CA) change under the fed-batch fermentation by the engineered *Y. lipolytica* Ylxs48 in the 100 L bioreactor containing an initial 40 L of YPNP medium supplemented with 310 g/L glucose. More glucose (3,200 g, 3,200 g, and 2,400 g) was added to the bioreactor in triplicate at 4 L, 4 L, and 2 L each.

Under 100 L fed-batch fermentation, the process was started as a batch culture with an initial glucose concentration of 310 g/L. More glucose (3,200, 3,200, 2,400 g) was added to the bioreactor in triplicate at 4, 4, and 2 L volume each, resulting in a total glucose concentration of 424 g/L (total volume was 50 L). Figure 6 shows in more detail the representative culture with three glucose pulses. The viable cell density of engineered strain Ylxs48 reached a maximum of 8.09 pF/cm at 14 h and only slightly decreased to 7.39 pF/cm before the first glucose pulse (Fig. 6A). It remained stable at every stage after feeding. Within the 24 h, the total biomass increased to OD_600_ 33. After 24 h fermentation, the pH drops from 6.77 to 3.14 (Fig. 6A and D) due to the accumulation of 5.71 g/L CA (Fig. 6F) in the culture broth from cell primary metabolism. The DO also dropped from 100% to 2.71% within 12 h, and it gradually increased after 24 h (Fig. 6A). Erythritol synthesis achieved a yield of 0.38 g/g glucose from 0 to 24 h, indicating that only a part of the glucose was utilized for biomass creation. The biomass reached the maximal OD_600_ value of 37.12 (9.28 g/L DCW) at 40 h. It did not increase further over time, suggesting thus that all glucose fed to the bioreactor was used for cell viability maintenance and erythritol synthesis. The OUR at this point reached a maximum of 53.57 mmol/(L⋅h). Maximum CER at the same time was at 69.55 mmol/(L⋅h), and the average RQ during the erythritol production phase was 1.35 (Fig. 6B). The rate of glucose consumption gradually increased, reaching a maximum value of 9.14 g/(L·h) between 36 and 40 h, while the yield of erythritol reached 0.77 g/g glucose (Fig. 6E). After the first glucose pulse, the yield between 40 and 50 h reached a maximum of 0.78 g/g glucose (Fig. 6E). After 50 h, both glucose consumption rate and erythritol productivity started to decrease. From 36 to 50 h, the glucose consumption rate was stable at 9.15–9.21 g/(L·h), while the erythritol productivity was at 7.06–7.17 g/(L·h). The productivity between 60 and 74 h reduced to 4.48 g/(L·h), 1.7-fold higher than that of the control strain batch fermentation [2.63 g/(L·h)]. The higher yield was achieved by extending the effective erythritol production duration, as a result of the higher erythritol productivity for the strain Ylxs48 than that of the parental strain Ylxs01. The increased productivity also reduced the proportion of glucose consumed by aerobic respiration throughout the fermentation process (Table S10). This phenomenon was consistent with previous studies showing that the yield of erythritol ranged between 0.45 and 0.83 g/g glycerol under the repeated batch culture conducted for *Y. lipolytica* MK1 (12).

Under 200 L fed-batch fermentation, the process was started as a batch culture with an initial glucose concentration of 310 g/L. After glucose depletion, the culture was fed with three doses of 11.2 kg for the first two times and 8.4 kg for the third by using glucose dry powder, resulting in a total glucose concentration of 530 g/L (total volume was 140 L). Due to the fermentation medium being acidic (pH <3.0), high osmotic pressure (>2.0 osmol/kg) and high cell density (OD_600_ >35), which allowed direct feeding of high concentration of glucose without sterilization and avoided the risk of contamination. Glucose (74.2 kg) was completely consumed after 83 h, and the highest erythritol titer of 355.81 g/L was obtained. The erythritol yield (0.71 g/g glucose) obtained in scale-up fermentation had no significant difference compared with that of in the 3 L bioreactor (0.72 g/g). No additional polyol by-products (mannitol and arabitol) were produced (Fig. S6), which is beneficial to the subsequent purification of erythritol. Based on the above results, it was determined that the engineered *Y. lipolytica* strain Ylxs48 was robust and showed excellent performance in the pilot scale-up fermentation process.

The KPI parameters of other yeasts for erythritol production were summarized in Table 3. The yeast *Pseudozyma tsukubaensis* produced 241 g/L erythritol when grown in a medium containing glucose, which provided the highest productivity of 2.84 g/(L⋅h) among the reported results (32). The reported highest yield was 0.67 g/g glycerol under repeated batch fermentation by *Y. lipolytica* MK1 (12). Compared with other strains, the engineered *Y. lipolytica* strain Ylxs48 used in this study demonstrated superior advantages in all aspects. Notably, it exhibited a remarkable increase in productivity, reaching 4.62 and 4.29 g/(L⋅h) under batch and fed-batch conditions, respectively. This will help shorten the process duration and reduce the cost associated with higher erythritol production. Because in the process of erythritol production, the cost is strongly correlated with the fermentation time (14).

### General techno-economic analysis of the erythritol production process for the engineered strain Ylxs48

A cost analysis of erythritol production for strains Ylxs01 and Ylxs48 was performed based on data collected at a 200 L pilot scale. The production cost employing both strains varied in terms of energy costs (including electricity and steam costs), raw material glucose, and labor. Considering that the medium composition remained similar, and the biomass did not undergo major changes, there were no noticeable differences in other expenses, such as ion exchange, nitrogen supply, and decolorization costs. Therefore, we conducted a thorough comparison and analysis of the costs associated with energy sources, glucose, and labor. Owing to the significant reduction in process duration, the batch fermentation time has been shortened from an average of 72 to 46 h, with an average reduction of 26 h, resulting in a reduction of approximately 30% in electricity costs. Using the control strain Ylxs01, the electricity consumption per 1,000 kg of erythritol product was about 2,300 kWh. According to the electricity price of 0.1 USD/kWh, the electricity cost per 1,000 kg was 230 USD. After using the engineered strain Ylxs48, the electricity consumption per 1,000 kg of product was 1,610 kWh, and the cost of electricity per 1,000 kg was $161, saving $69 in electricity costs per 1,000 kg of product.

The concentration of erythritol in the fermentation broth increased from an average of 173 to 213 g/L. In industrial production, it is generally necessary to use high-temperature steam to concentrate erythritol in the fermentation broth to 650 g/L for crystallization. The former has a concentration factor of 3.8 (650/173), while the latter has a concentration factor of 3.1 (650/213), thus saving approximately 20% of the steam required for the concentration. Using the strain Ylxs01 for fermentation, the steam cost per 1,000 kg of erythritol product is approximately $115, while using the new strain Ylxs48 reduces the steam cost per 1,000 kg of product to $92, a reduction of $23.

With strain Ylxs48, the conversion rate (yield) of glucose to erythritol increased from 0.55 to 0.69 g/g glucose. The synthesis of 1,000 kg erythritol by using the control strain Ylxs01 necessitated the consumption of 2,000 kg glucose monohydrate. Based on a price of $500 per 1,000 kg of glucose monohydrate, the price of 2,000 kg of glucose monohydrate was $1,000. By using the engineered strain Ylxs48, synthesis of 1,000 kg of erythritol required approximately 1,600 kg, with a price of $800. Therefore, by adopting the engineered strain Ylxs48, the cost of glucose decreased by approximately $200.

With strain Ylxs48, the productivity has increased by 92.5% [i.e., from 2.4 to 4.62 g/(L⋅h)], resulting in a corresponding reduction in labor costs. The labor cost per 1,000 kg of erythritol was approximately $45. When using the engineered strain Ylxs48, the labor cost was $23, a decrease of $22.

According to the current average production cost of erythritol per 1,000 kg in China, which was approximately 1,800 USD, the adoption of the engineered strain Ylxs48 resulted in a total cost reduction of 314 USD. Consequently, the total cost amounts to 1,486 USD, reflecting a reduction of 17.4%. If the concentration of erythritol was increased to 355.81 g/L after three consecutive glucose fed-batch, it would further reduce the steam cost required for concentration. Similarly, the cost of producing 1,000 kg of erythritol will be further reduced to $1,386, a decrease of 23%, with a significant competitive advantage.

Table 4 showed the general cost analysis of erythritol production using the engineered strain YLxs48, with the total erythritol production cost reduced by 17.4% and 23% when batch and fed-batch were employed separately. Owing to a significant improvement in conversion rate (by 15%), more glucose is used to synthesize erythritol, thereby reducing carbon emissions by about 15%.

**TABLE 4:** General cost analysis of per 1,000 kg erythritol production (USD)^*a*^

Cost type	Result for the following strain/strategy:		
	Ylxs01 Batch	Ylxs48 Batch	Ylxs48 Fed-batch
Electricity cost	230	161	140
Steam cost	115	92	60
Glucose cost	1,000	800	750
Labor cost	45	23	26
Other costs	410	410	410
Total costs	1,800	1,486	1,386
Reduction cost	–^*b*^	314	414
Reduction rate	–	17.40%	23.00%

^*a*^ Other costs, including nitrogen, ion exchange, decolorization, quality check, packaging, etc., are of no difference when using the two strains.

^*b*^ –, not applicable.

### Conclusions

For the first time, the combination of hexose transport and pathway engineering was applied to the metabolic modification of erythritol-producing industrial *Y. lipolytica*. As a result, the erythritol production time in batch fermentation was successfully reduced to 46 h, which was 26 h faster than that of the parental strain. The high efficiency of the engineered *Y. lipolytica* using glucose allowed for three glucose feedings in 83 h, resulting in erythritol yield, titer, and productivity of 0.71 g/g glucose, 355.81 g/L, and 4.29 g/(L⋅h) in 200 L bioreactors. Erythritol can be obtained by direct crystallization from the clear supernatant at 4°C after secondary glucose feeding. This eliminates the need for excessive high-temperature steam in the subsequent purification procedure, which involves the evaporation concentration. It is attractive to further improve the performance of industrial strains, while combining substrate transport and endogenous synthetic pathways is an effective approach. The strategy proposed here will further reduce the production cost of erythritol and provide a new idea for the performance optimization of other industrial strains.

## Data Availability

The data that support the findings of this study are available on request from the corresponding author.

## SUPPLEMENTAL MATERIAL

Supplemental material aem.00061-25-s0001.docx Tables S1 to S10, Figures S1 to S6, and supplemental methods.

ASM does not own the copyrights to Supplemental Material that may be linked to, or accessed through, an article. The authors have granted ASM a non-exclusive, world-wide license to publish the Supplemental Material files. Please contact the corresponding author directly for reuse.
