# Supplementary material for: Synergistic effect of transporter and pathway engineering on the key performance indicators of erythritol synthesis by the yeast Yarrowia lipolytica
Source: Appl Environ Microbiol. 2025 Mar 26;91(4):e00061-25. doi: 10.1128/aem.00061-25 (PMC12016529; doi:10.1128/aem.00061-25)
Supplement: Supplemental material — Tables S1 to S10, Figures S1 to S6, and supplemental methods. [file aem.00061-25-s0001.docx]

**Supporting Information**

**Synergistic effect of transporter and pathway engineering on the key performance indicators of erythritol synthesis by the yeast *Yarrowia lipolytica***

Shuo Xu ^a^, Qian Li ^a^, Ye Li ^a^, Yue Zhang ^a^, Qing Li ^a^, Liyun Ji ^a^, Hairong Cheng ^a*^

*^a^ State Key Laboratory of Microbial Metabolism, and School of Life Sciences and Biotechnology, Shanghai Jiao Tong University, Shanghai 200240, China.*

* Corresponding author: Hairong Cheng

Email: chrqrq@sjtu.edu.cn

**Contents:**

**Supplementary Table S1-S10:**

**Table S1.** Genes overexpressed in *Y. lipolytica*.

**Table S2.** Plasmids used in this study.

**Table S3.** Sequences of primers used for site-specific homologous recombinant plasmids construction.

**Table S4.** Sequences of primers used for construction of the single gene screening strains.

**Table S5.** Sequences of primers used for pathway and transporter engineering.

**Table S6.** Sequences of primers used for Real-Time PCR.

**Table S7.** Sequences of *Vhb* and *SpHXK1*.

**Table S8.** Relative growth rate and glucose uptake rate (Q_GLU_) of the engineered *Y.lipolytica* strains.

**Table S9.** Key performance indicators of erythritol synthesis by the parental and engineered *Y.lipolytica* strains under 100 L batch and glucose fed-batch conditions.

**Table S10.** Distribution of glucose consumption by the parental and engineered *Y.lipolytica* strains under 100 L batch and glucose fed-batch conditions.

**Supplementary Figure S1-S6:**

**Figure S1.** Schematic diagram of the strain construction process.

**Figure S2.** The microscopy images of the Ylxs01, Ylxs30, and Ylxs31 grown on YNB plates for 4 days.

**Figure S3.** Chromatographic diagram of substrates and products analysis of the control strain Ylxs01 and the engineered *Y.lipolytica* Ylxs48.

**Figure S4.** Comparison of cell activity of the control strain Ylxs01 and the engineered *Y.lipolytica* Ylxs48 at different fermentation times.

**Figure S5.** Schematic diagram of the glucose batch fermentation process of the parental strain Ylxs01 and the engineered strain Ylxs48 in the 100 L bioreactor.

**Figure S6.** Chromatographic diagram of polyol products analysis of the control strain YlxsWT and the engineered *Y. lipolytica* Ylxs48.

**Methods.** Construction of homologous recombinant integrative plasmids.

**Supplementary references**

**Table S1.** Genes overexpressed in *Y. lipolytica.*

| **Genes ID** | **Name** | **Enzyme encoded** | **Origin** |
| --- | --- | --- | --- |
| YALI0_E22649g | *ZWF1* | Glucose-6-phosphate dehydrogenase | *Y. lipolytica* |
| YALI0_B15598g | *GND1* | 6-Phosphogluconate dehydrogenase | *Y. lipolytica* |
| YALI0_C11880g | *RPE1* | Ribulose-5-phosphate epimerase | *Y. lipolytica* |
| YALI0_B06941g | *RPI1* | Ribulose-5-phosphate isomerase | *Y. lipolytica* |
| YALI0_E06479g | *TKL1* | Transketolase | *Y. lipolytica* |
| YALI0_F15587g | *TAL1* | Transaldolase | *Y. lipolytica* |
| YALI0_F07711g | *PGI1* | Glucose-6-phosphate isomerase | *Y. lipolytica* |
| YALI0_D16357g | *PFK1* | Phosphofructokinase | *Y. lipolytica* |
| YALI0_E26004g | *FBA1* | Fructose bisphosphate aldolase | *Y. lipolytica* |
| YALI0_F05214g | *TPI1* | Triose-phosphate isomerase | *Y. lipolytica* |
| YALI0_D07634g | *ER10* | Erythrose reductase | *Y. lipolytica* |
| YALI0_B07117g | *ER16* | Erythrose reductase | *Y. lipolytica* |
| YALI0_C13508g | *ER25* | Erythrose reductase | *Y. lipolytica* |
| YALI0_F18590g | *ER27* | Erythrose reductase | *Y. lipolytica* |
| YALI0_C06424g | *YHT1* | Hexose transporter | *Y. lipolytica* |
| YALI0_F19184g | *YHT3* | Hexose transporter | *Y. lipolytica* |
| YALI0_E23287g | *YHT4* | Hexose transporter | *Y. lipolytica* |
| YALI0_B01342g | *YHT5* | Hexose transporter | *Y. lipolytica* |
| YALI0_E15488g | *GLK1* | Hexokinase | *Y. lipolytica* |
| YALI0_B22308g | *HXK1* | Hexokinase | *Y. lipolytica* |
| YALI0_C10098g | *DID2* | A subunit of the endosomal sorting complex | *Y. lipolytica* |
| YALI0_E11495g | *AMPD* | AMP deaminase | *Y. lipolytica* |
| YALI0_D02101g | *Snf1* | Sucrosenon-fermenting protein kinase | *Y. lipolytica* |
| NP_592948.1 | *SpHXK1* | Hexokinase | *Schizosaccharomyces pombe* |
| BDX35413.1 | *Vhb* | Bacterial hemoglobin | *Vitreoscilla stercoraria* |
| YNL010W | *PYP1* | Polyol phosphatase 1 | *Saccharomyces cerevisiae* |
| SCEN_D00160 | *ScTHI13* | 4-Amino-5-hydroxymethyl-2-methylpyrimidine phosphate synthase | *S. cerevisiae* |

**Table S2.** Plasmids used in this study.

| **Plasmids** | **description** | **reference** |  |
| --- | --- | --- | --- |
| pSWV-*hph* | *Amp*^r^ *hph* | (1) |  |
| pXS-*guaB* | *Amp*^r^ *guaB,* P_hp4d_ | (2) |  |
| pUra3-updw | *Amp*^r^, *ura3* site-specific integrated vector |  |  |
| pMhy1-*guaB* | *Amp*^r^ *guaB*, *MHY1*:: *guaB* |  |  |
| pCla4-*guaB* | *Amp*^r^ *guaB,* *CLA4*:: *guaB* |  |  |
| pIntA-*guaB* | *Amp*^r^ *guaB*, intA site-specific integrated expression vector |  |  |
| pIntB-*guaB* | *Amp*^r^ *guaB*, intB site-specific integrated expression vector |  |  |
| pIntC1-*guaB* | *Amp*^r^ *guaB*, intC1 site-specific integrated expression vector |  |  |
| pIntC3-*YHT1/3* | Cloning *YHT1* and *YHT3* gene into pIntC3-*guaB* |  |  |
| pExp1-*guaB* | *Amp*^r^ *guaB,* P_exp1_ |  |  |
| pSnf1-*guaB* | *Amp*^r^ *guaB,* *Snf1*:: *guaB* | This work |  |
| pTef1-*guaB* | *Amp*^r^ *guaB,* P_tef1_ | This work |  |
| pGpd1-*guaB* | *Amp*^r^ *guaB,* P_gpd1_ | This work |  |
| pTef1-*ZWF1* | Cloning P_tef1_-*ZWF1* gene into pIntA-*guaB* | This work |  |
| pIntA-*ZWF1* | Cloning *ZWF1* gene into pIntA-*guaB* | This work |  |
| pIntA-*GND1* | Cloning *GND1* gene into pIntA-*guaB* | This work |  |
| pIntA-*RPE1* | Cloning *RPE1* gene into pIntA-*guaB* | This work |  |
| pIntA-*RPI1* | Cloning *RPI1* gene into pIntA-*guaB* | This work |  |
| pIntA-*TKL1* | Cloning *TKL1* gene into pIntA-*guaB* | This work |  |
| pIntA-*TAL1* | Cloning *TAL1* gene into pIntA-*guaB* | This work |  |
| pIntA-*PGI1* | Cloning *PGI1* gene into pIntA-*guaB* | This work |  |
| pIntA-*PFK1* | Cloning *PFK1* gene into pIntA-*guaB* | This work |  |
| pIntA-*FBA1* | Cloning *FBA1* gene into pIntA-*guaB* | This work |  |
| pIntA-*TPI1* | Cloning *TPI1* gene into pIntA-*guaB* | This work |  |
| pIntA-*ER10* | Cloning *ER10* gene into pIntA-*guaB* | This work |  |
| pIntA-*ER16* | Cloning *ER16* gene into pIntA-*guaB* | This work |  |
| pIntA-*ER25* | Cloning *ER25* gene into pIntA-*guaB* | This work |  |
| pIntA-*ER27* | Cloning *ER27* gene into pIntA-*guaB* | This work |  |
| pIntA-*YHT1* | Cloning *YHT1* gene into pIntA-*guaB* | This work |  |
| pIntA-*YHT3* | Cloning *YHT3* gene into pIntA-*guaB* | This work |  |
| pIntA-*YHT4* | Cloning *YHT4* gene into pIntA-*guaB* | This work |  |
| pIntA-*YHT5* | Cloning *YHT5* gene into pIntA-*guaB* | This work |  |
| pIntA-*GLK1* | Cloning *GLK1* gene into pIntA-*guaB* | This work |  |
| pIntA-*HXK1* | Cloning *HXK1* gene into pIntA-*guaB* | This work |  |
| pIntA-*SpHXK1* | Cloning *SpHXK1* gene into pIntA-*guaB* | This work |  |
| pIntA-*AMPD* | Cloning *AMPD* gene into pIntA-*guaB* | This work |  |
| pIntA-*DID2* | Cloning *DID2* gene into pIntA-*guaB* | This work |  |
| pIntA-*PYP1* | Cloning *PYP1* gene into pIntA-*guaB* | This work |  |
| pIntA-*Vhb* | Cloning *Vhb* gene into pIntA-*guaB* | This work |  |
| pIntA-*ScTHI13* | Cloning P_3_-*ScTHI13* gene into pIntA-*guaB* | This work |  |
| pMhy1-*TKL1* | Cloning *TKL1* gene into pMhy1-*guaB* | This work |  |
| pCla4-*TAL1* | Cloning *TAL1* gene into pCla4-*guaB* | This work |  |
| pIntB-*HXK1* | Cloning *HXK1* gene into pIntB-*guaB* | This work |  |
| pIntC1-*YHT4* | Cloning *YHT4* gene into pIntC1-*guaB* | This work |  |
| pExp1-*ER27* | Cloning *ER27* gene into pExp1-*guaB* | This work |  |
| pExp1-*SpHXK1* | Cloning *SpHXK1* gene into pExp1-*guaB* | This work |  |
| pExp1-*TAL1* | Cloning *TAL1* gene into pExp1-*guaB* | This work |  |
| pExp1-*Vhb* | Cloning *Vhb* gene into pExp1-*guaB* | This work |  |
| pExp1-*YHT5* | Cloning *YHT5* gene into pExp1-*guaB* | This work |  |
| pGpd1-*PGI1* | Cloning *PGI1* gene into pGpd1-*guaB* | This work |  |
| pTef1-*PFK1* | Cloning *PFK1*gene into pTef1-*guaB* | This work |  |
| pTef1-*GLK1* | Cloning *GLK1* gene into pTef1-*guaB* | This work |  |
| pTef1-*GND1* | Cloning *GND1* gene into pTef1-*guaB* | This work |  |
| pIntC2-updw | *Amp*^r^, intC2 site-specific integrated vector | This work |  |
| pIntD1-updw | *Amp*^r^, intD1 site-specific integrated vector | This work |  |
| pIntE2-updw | *Amp*^r^, intE2 site-specific integrated vector | This work |  |
| pIntE3-updw | *Amp*^r^, intE3 site-specific integrated vector | This work |  |
| pIntE4-updw | *Amp*^r^, intE4 site-specific integrated vector | This work |  |
| pIntF2-updw | *Amp*^r^, intF2 site-specific integrated vector | This work |  |
| pIntF3-updw | *Amp*^r^, intF3 site-specific integrated vector | This work |  |
| pIntC2-*GLK1/SpHXK1* | Cloning *GLK1*, *SpHXK1* and *guaB* genes into pIntC2-updw | This work |  |
| pIntD1-*PGI1/ER16* | Cloning *PGI1*, *ER16* and *guaB* genes into pIntD1-updw | This work |  |
| pIntE2-*GND1/ER25* | Cloning *GND1*, *ER25* and *guaB* genes into pIntE2-updw | This work |  |
| pIntE2-*PFK1/FBA1* | Cloning *PFK1*, *FBA1* and *guaB* genes into pIntE2-updw | This work |  |
| pIntE3-*PYP1/ER27* | Cloning *PYP1*, *ER27* and *guaB* genes into pIntE3-updw | This work |  |
| pIntE4-*RPI1/TAL1* | Cloning *RPI1*, *TAL1* and *guaB* genes into pIntE4-updw | This work |  |
| pIntF2-*ScTHI13/ER10* | Cloning *ScTHI13*, *ER10* and *guaB* genes into pIntF2-updw | This work |  |
| pIntF3-*DID2/Vhb* | Cloning *DID2*, *Vhb* and *guaB* genes into pIntF3-updw | This work |  |
| pUra3-*GLK1/TPI1* | Cloning *GLK1*, *TPI1* and *guaB* genes into pUra3-updw | This work | |
| pIntC1-*YHT4/5* | Cloning *YHT4*, *YHT5* and *guaB* genes into pIntC1-*guaB* | This work | |

**Table S3.** Sequences of primers used for site-specific homologous recombinant plasmids construction.

| **Primers** | **Sequence 5′→3′** |
| --- | --- |
| Snf1-up-F | CGTCTCTTTATGCGGCCGCGAATTCTGGATCACTACGAAGCTGAATGAGC |
| Snf1-up-R | GTTGAGGGGATCCGCAGAGTTCAATCGAGAAAACGCG |
| Snf1-dw-F | CTCTGCGGATCCCCTCAACGTCAAGATTGCCGACT |
| Snf1-dw-R | GCAGTGACTCCGTCTCTGTAGAATTCCTTAGTTGTGCTGTCCGACTCATCC |
| Snf1-ck-f | CGATCAAGATCAAGATCTCCGACTGTC |
| Snf1-ck-r | TCTCCTTCATGAGGTCGTAGGC |
| Snf1-guaB-F | TTCTCGATTGAACTCTGCGGATCCGAATACGGTAGTCGCACGGACTT |
| Snf1-guaB-R | GCAATCTTGACGTTGAGGGGATCCCGATAAGGGTAGTGGTATTTCACCGG |
| Snf1g-ck-f | CCTGGCTCTATCTGTACTACTCGAATC |
| Snf1g-ck-r | TACAGCTTGCCACTGATAACCTCG |
| Y-snf1-ck-F | CAGAAATTAATCGCGCCATCATCTGTC |
| Y-snf1-ck-R | CCTTAAGTCCGTGCGACTACCGT |
| Snf1-K-F | TGGATCACTACGAAGCTGAATGAGC |
| Snf1-K-R | CTTAGTTGTGCTGTCCGACTCATCC |
| C2-up-F | CGTCTCTTTATGCGGCCGCGAATTCCTCCAGTCCAAATTCGACGACCTG |
| C2-up-R | GGGATCCGTCAAAACCAACCAGACCTCGGTAG |
| C2-dw-F | GTTGGTTTTGACGGATCCCATACAATCAGACGATTCGGTGCATC |
| C2-dw-R | GCAGTGACTCCGTCTCTGTAGAATTCCGATATTTTGAGTCTAGGGCGAATCCAT |
| C2-ck-f | CTACAAGCCGTCTAAGACTACACAAAGC |
| C2-ck-r | GACATGTGGCAATATCGTCAGACAG |
| C3-up-F | CGTCTCTTTATGCGGCCGCGAATTCCACACATCGGACCTTATGTCGCG |
| C3-up-R | CGGCTAACAAACGGGGATCCGTAAAAACCACCTGTTCATGGGCTAG |
| C3-dw-F | GGATCCCCGTTTGTTAGCCGCTTATATCGTTG |
| C3-dw-R | GCAGTGACTCCGTCTCTGTAGAATTCCTCTCGGGTCAGATTGATATATGCACTTG |
| C3-ck-f | CCACGCCGTCATAGATGCTACG |
| C3-ck-r | GTACCTTCCCCAGATCTCGCC |
| D1-up-F | CGTCTCTTTATGCGGCCGCGAATTCGATTAGTAATTCGGAGAACACATGAGCGC |
| D1-up-R | GGCCAGGGGATCCGACACAAACAGCAGACGCAGAC |
| D1-dw-F | GTGTCGGATCCCCTGGCCGGTTAAGTTAAACTCC |
| D1-dw-R | GCAGTGACTCCGTCTCTGTAGAATTCCCTAGACTCACAAGGTCACTTTTCCG |
| D1-ck-f | CAAAACTTCTCCAAAGCACGGGTAC |
| D1-ck-r | CTTTTCAGCGTCAACATTTTTCTCTCAGTT |
| E2-up-F | CGTCTCTTTATGCGGCCGCGAATTCGACAAGGCTCCAAACACTGACGAC |
| E2-up-R | CAAATGTCAAGCTTGGTGTCTTAGTGCAGCAAAATCGG |
| E2-dw-F | GACACCAAGCTTGACATTTGCAAAAGAAACGCGCTG |
| E2-dw-R | GCAGTGACTCCGTCTCTGTAGAATTCCGCCCTACGTCATAGTCTTGGC |
| E2-ck-f | CGTCAACTTTCTTGTCCGACCTCTC |
| E2-ck-r | GAGTGTAAACGGCCAAGTCTAGC |
| E3-up-F | CGTCTCTTTATGCGGCCGCGAATTCACGCCATTCTATAAGTGCAAGTGCTC |
| E3-up-R | CGAAGCGGATCCGTGATGAATTATAATTATGCACAGGTCCCAAATG |
| E3-dw-F | CATCACGGATCCGCTTCGTTCAATTCAGGTTTTAGGCATT |
| E3-dw-R | GCAGTGACTCCGTCTCTGTAGAATTCGGTGGGCCAATTACTTGTAGCTCTT |
| E3-ck-f | TACAGAACGCACAAACGGTACAC |
| E3-ck-r | TTGCATTTTGCCTCTTTGGGTCTC |
| E4-up-F | CGTCTCTTTATGCGGCCGCGAATTCGATATTATAGTGCTTCTGATCGCGCTAAGG |
| E4-up-R | TCACTCGGATCCGTTCTTTCTCCCCAAACTTCCTCTCTC |
| E4-dw-F | GAAAGAACGGATCCGAGTGAGAGGGACAGGATACACCG |
| E4-dw-R | GCAGTGACTCCGTCTCTGTAGAATTCCCAGAGCCCTAGTTTACGGCAAT |
| E4-ck-f | GCGGAGGAGCAATAGACATACGAT |
| E4-ck-r | GGCGTACCTTATTACGAGCTCCC |
| F2-up-F | CGTCTCTTTATGCGGCCGCGAATTCGCCAGCCCTTCAATTCTACTGCTT |
| F2-up-R | CCAAGTCCGGATCCTAGCCGTTCCCTAGTTATCTCCACC |
| F2-dw-F | GGCTAGGATCCGGACTTGGATCTGGGGTTTTATTCG |
| F2-dw-R | GCAGTGACTCCGTCTCTGTAGAATTCCTTTTCTCTCTGTCTGTTGCTGGTG |
| F2-ck-f | CCATCACCGTAACAACAATAGCAATGAC |
| F2-ck-r | GGACCATAAACTGCGATTAGTCAGC |
| F3-up-F | CGTCTCTTTATGCGGCCGCGAATTCGCTACTCCTCCCTAGATCTAAATCGCG |
| F3-up-R | GGATCCTAGCATCAACACTAATTCTCGGTCCC |
| F3-dw-F | GTGTTGATGCTAGGATCCCATCTACCGCAGCTACTATACACGAG |
| F3-dw-R | GCAGTGACTCCGTCTCTGTAGAATTCGAAAGTCCAAATGCGATGGTCACG |
| F3-ck-f | CAAGTTTCAGACTTGCTCCTTTTGAGTC |
| F3-ck-r | TCGTCGCCCAACAAGTGCAAC |

**Table S4.** Sequences of primers used for construction of the single gene screening verification strains.

| **Primers** | **Sequence 5′→3′** |
| --- | --- |
| TEF-Z-F | CCATACTCAAGCTTGTCGACACTAGTGAAGTCGACCAGAGACCGGGTTG |
| TEF-Z-R | GCCAGTCATTTTGAATGATTCTTATACTCAGAAGGAAATGCTT |
| T-ZWF1-F | CATTCAAAATGACTGGCACCTTACCCAAGTTC |
| T-ZWF1-R | TATCTGTTAATTGCCTGCAGTCACGAGGAGCCCTTGGTGAC |
| T-Z-ck-f | CTGCAGTCTGGAATCTACGCTTG |
| T-Z-ck-r | GTCTCGGATGATTCCAATGTCGTTG |
| ZWF1-F | CCCGAAACTAAGCATATGGGTACCATGACTGGCACCTTACCCAAGTTC |
| ZWF1-R | TATCTGTTAATTGCCTGCAGGGTACCTCACGAGGAGCCCTTGGTGAC |
| ZWF1-ck-f | GACATTTCCTGGAAGATTTTCACCGATC |
| ZWF1-ck-r | CAGAGCTTCTTTAGCGATTCGGAG |
| GND1-F | CCCGAAACTAAGCATATGGGTACCATGACTGACACTTCAAACATCAAGCC |
| GND1-R | TATCTGTTAATTGCCTGCAGGGTACCTTAAGCATCGTAAGTGGAAGAAGAAACC |
| GND1-ck-f | CCCGAGACATCTTCAAGTACGACG |
| GND1-ck-r | GTATTCGAACACGGGCATCTCAC |
| RPE1-F | CCCGAAACTAAGCATATGGGTACCATGGTCCAGCCAATCATCGCC |
| RPE1-R | TATCTGTTAATTGCCTGCAGGGTACCTTAGGCAGAGGCAGCAGCGG |
| RPE1-ck-f | TTCTTTCCTCAGACTTTGCCAAGC |
| RPE1-ck-r | GTATTCGAACACGGGCATCTCAC |
| RPI1-F | CCCGAAACTAAGCATATGGGTACCATGTCCTCCGAACTGCCTCCTC |
| RPI1-R | TATCTGTTAATTGCCTGCAGGGTACCTTACTTGGTAATGGTGGAGATGGTTCC |
| RPI1-ck-f | CCTCCGAACTGCCTCCTCTTG |
| RPI1-ck-r | ATTCGAACACGGGCATCTCACT |
| TKL1-F | CCCGAAACTAAGCATATGGGTACCATGGCTCCCCAATTTTCAAAGACTGAC |
| TKL1-R | TATCTGTTAATTGCCTGCAGGGTACCTTAGACACCGTGGCCGGGTCG |
| TKL1-ck-F | GCAAACCTGACCCGATGGAAG |
| TKL1-ck-R | GGTTAAGAGAATTATCACCGGCAAAC |
| TAL1-F | CCCGAAACTAAGCATATGGGTACCATGTCTTCCAACTCTCTTGAACAGCT |
| TAL1-R | TATCTGTTAATTGCCTGCAGGGTACCCTAAGCGGAGAGCTTGGTCTCAATG |
| TAL1-ck-f | GTACGCCAAGCTCATTGACGAGG |
| TAL1-ck-r | CGAACAAAGACGGGATTTTGCCAC |
| PGI1-F | CCCGAAACTAAGCATATGGGTACCATGGCTCAGTCCTTCACGACC |
| PGI1-R | TATCTGTTAATTGCCTGCAGGGTACCTCAAGCGGCCCAAGCCTTGT |
| PGI1-ck-f | GTTTCCTTTTGCCTCTTGGGTTGG |
| PGI1-ck-r | GGGATTTTGCCACCTACAAGCC |
| PFK1-F | CCCGAAACTAAGCATATGGGTACCATGATTGAAGGAATCTCCTTTGCGTC |
| PFK1-R | TATCTGTTAATTGCCTGCAGGGTACCCTAACAAGGATCAATAATACCCTGCTCC |
| PFK1-ck-f | CCATTTCGAACAACGTGCCTGG |
| PFK1-ck-r | GTATTCGAACACGGGCATCTCAC |
| FBA1-F | CCCGAAACTAAGCATATGGGTACCATGCCTGTTACTGACGTCCTTAAGC |
| FBA1-R | TATCTGTTAATTGCCTGCAGGGTACCTTACAAGGTGTTCTTGGCGTTGAAAAC |
| FBA1-ck-f | CAAGGATCAGACCGCCTCCATC |
| FBA1-ck-r | GGATTTTGCCACCTACAAGCCAG |
| TPI1-F | CCCGAAACTAAGCATATGGGTACCATGTCTCGAACCTTTTTTGTTGGCG |
| TPI1-R | TCTGTTAATTGCCTGCAGGGTACCTTAAAGTCGAGAGTTGATGATGTCAACAAAC |
| TPI1-ck-f | GGAAACTTCAAGATGAACGGCTCTC |
| TPI1-ck-r | GTATTCGAACACGGGCATCTCAC |
| ER10-F | CCCGAAACTAAGCATATGGGTACCATGTCCTTCAAGCTCGCCTCCG |
| ER10-R | TATCTGTTAATTGCCTGCAGGGTACCTTAGGCGAAAATGGGAAGGTTAGC |
| ER10-ck-f | GCCATCAAGAACGGTTACAGACTG |
| ER10-ck-r | GGATTTTGCCACCTACAAGCCAG |
| ER16-F | CCCGAAACTAAGCATATGGGTACCATGTTCCGGTCAGTATATAAACGGGG |
| ER16-R | TATCTGTTAATTGCCTGCAGGGTACCTTAGCAGAAGTCAAAGTCGGGGAAT |
| ER16-ck-f | CCGCCTTCAACTACCGAAACG |
| ER16-ck-r | CGGGATTTTGCCACCTACAAGC |
| ER25-F | CCCGAAACTAAGCATATGGGTACCATGCCAATTATCACAGAAACATTCAAGC |
| ER25-R | TCTGTTAATTGCCTGCAGGGTACCCTATTTGTCCTCGAAACCAAGGTTGTC |
| ER25-ck-F | ACCGCCTACATCTACAAATCCG |
| ER25-ck-R | GATTTTGCCACCTACAAGCCAG |
| ER27-F | CCCGAAACTAAGCATATGGGTACCATGGCAGGCGGACCCACTCTC |
| ER27-R | TATCTGTTAATTGCCTGCAGGGTACCTTACTTCTTCTGCTCAGCAAGGTACTTC |
| ER27-ck-f | AAACTGGAACAACGCCCGAAC |
| ER27-ck-r | ATTCGAACACGGGCATCTCAC |
| YHT1-F | CCCGAAACTAAGCATATGGGTACCATGGGACTCGCTAACATCATCAACC |
| YHT1-R | TATCTGTTAATTGCCTGCAGGGTACCCTAGACAGACTCAATGTAGACGTGCTG |
| YHT1-ck-f | CTCATCCTGCTACCGGAGACTC |
| YHT1-ck-r | GTGGGAGGTTAAGAGAATTATCACCGG |
| YHT3-F | CCCGAAACTAAGCATATGGGTACCATGTCCACTAGTGCTATGACCGAC |
| YHT3-R | TATCTGTTAATTGCCTGCAGGGTACCCTAAGAGGACTCGGAGAAGTCGG |
| YHT3-ck-f | CCTTGGTATTCTCGTCCAGGTTCTC |
| YHT3-ck-r | GTATTCGAACACGGGCATCTCAC |
| YHT4-F | CCCGAAACTAAGCATATGGGTACCATGGCGAGGCTTTGTCTTTCTC |
| YHT4-R | TATCTGTTAATTGCCTGCAGGGTACCTTAAACAGTCTCGGTGTACTGAGGAT |
| YHT4-ck-f | CAGCTCACCGGAATGAACGTC |
| YHT4-ck-r | GGATTTTGCCACCTACAAGCCAG |
| YTH5-F | CCCGAAACTAAGCATATGGGTACCATGTACAAGGTCCATAACCCCTACC |
| YTH5-R | TATCTGTTAATTGCCTGCAGGGTACCTTAGACATGCTCAGTTCCAGGATACTG |
| YTH5-ck-f | GAAAAGACTCCATCAACCGAACCATG |
| YTH5-ck-r | CGGGATTTTGCCACCTACAAGC |
| GLK1-F | CCCGAAACTAAGCATATGGGTACCATGACAATCACTCTGAGTCAGAAGGTTG |
| GLK1-R | TATCTGTTAATTGCCTGCAGGGTACCCTATGAGTCGTCCGGGTAAGCAG |
| GLK1-ck-f | GAGCTTACGCCTCTAAATCCGACG |
| GLK1-ck-r | GGATTTTGCCACCTACAAGCCAG |
| ylHXK1-F | CCCGAAACTAAGCATATGGGTACCATGGTTCATCTTGGTCCCCGAAAAC |
| ylHXK1-R | TATCTGTTAATTGCCTGCAGGGTACCCTAAATATCGTACTTGACACCGGGC |
| ylHXK1-ck-f | CTCCAACTACCACGACCCCC |
| ylHXK1-ck-r | GGATTTTGCCACCTACAAGCCAG |
| spHXK1-F | CCCGAAACTAAGCATATGATGTCCCTCCACGACGCTTAC |
| spHXK1-R | TATCTGTTAATTGCCTGCAGTTAATTCTTGAGATGCTCGGCGAGAATAT |
| spHXK1-ck-f | CGACTCAATGCCATTCTCTCCGAT |
| spHXK1-ck-r | GACGGGATTTTGCCACCTACAAG |
| AMPD-F | CCCGAAACTAAGCATATGGGTACCATGCCGCAGCAAGCAATGGAT |
| AMPD-R | TATCTGTTAATTGCCTGCAGTTAACCATGCAGCCGCTCAAAC |
| AMPD-ck-f | TTTTCGAGGTCACCAAGGATCCC |
| AMPD-ck-r | CGGGATTTTGCCACCTACAAGC |
| DID-F | CCCGAAACTAAGCATATGGGTACCATGTCTGGGCTCGAAAAGTCGTTG |
| DID-R | TATCTGTTAATTGCCTGCAGTTACCCCCTCAGAGCTCGCAAT |
| DID-ck-f | CGTCAGACTAACAGTGCCATTACAG |
| DID-ck-r | GGGATTTTGCCACCTACAAGCC |
| PYP1-F | CCCGAAACTAAGCATATGATGGTCAAAGCTGTTATTTTTACCGATTTCG |
| PYP1-R | TATCTGTTAATTGCCTGCAGCTAATTTTCCATCAATTCAGCGACTGTCTT |
| PYP1-ck-f | ACGATTATCTGACCGATACTTTAGGTTTCG |
| PYP1-ck-r | GTATTCGAACACGGGCATCTCAC |
| Vhb-F | CCCGAAACTAAGCATATGGGTACCATGCTCGACCAGCAAACCATTAAC |
| Vhb-R | TATCTGTTAATTGCCTGCAGGGTACCTTATTCAACGGCTTGAGCGTAGAGATC |
| Vhb-ck-f | CAAACCATTAACATCATCAAAGCCACTG |
| Vhb-ck-r | GGATCCCTTAAGTCCGTGCGAC |
| P3-F | CCATACTCAAGCTTGTCGACACTAGTGAGGGGTAGTCGTAAGTTTCATCACG |
| P3-R | CTGTAGACATGTTAATTGTAGGTGATATAAGGGGAAGGGT |
| P3-THI13-F | CTACAATTAACATGTCTACAGACAAGATCACATTTTTGTTG |
| P3-THI13-R | TATCTGTTAATTGCCTGCAGTTAAGCTGGAAGAGCCAATCTCTTGAAAG |
| PT-ck-f | CTGGATCCCCTTGTTACGTCCAC |
| PT-ck-r | GGCAGAATGGCTAATCTCTTACCG |

**Table S5.** Sequences of primers used for pathway and transporter engineering construction.

| **Primers** | **Sequence 5′→3′** | | |
| --- | --- | --- | --- |
| GPD-F | CGGACCCGCAAGCTTGTCGACGACGCAGTAGGATGTCCTGCACG | | |
| GPD-R | GTTAATTGCCTGCAGGGTACCTGTTGATGTGTGTTTAATTCAAGAATGAATATAG | | |
| GPD-ck-f | GGTTGAAATTCCGGCACTTGGATG | | |
| GPD-ck-r | CTTAAAAAGTGGCCTCCCAACACC | | |
| TEF-F | GCAGCCGGACCCGCAAGCTTGTCGACAGAGACCGGGTTGGCGGCGC | | |
| TEF-R | GAGGTACCTTTGAATGATTCTTATACTCAGAAGGAAATGCTTAACGATTTC | | |
| CYC1-F | TAAGAATCATTCAAAGGTACCTCATGTAATTAGTTATGTCACGCTTACATTCAC | | |
| CYC1-R | GAAGTTATGCTAGCGGATCCCTTAAGCGAGCGTCCCAAAACCTTCTC | | |
| TEF-ck-f | CAGATCTTGGTGGTAGTAGCAAATATTCAAATG | | |
| TEF-ck-r | TGCTTAACGATTTCGGGTGTGAG | | |
| exp-ER27-F | CACAAGACATATCTACAGCAGGTACCATGGCAGGCGGACCCACTCTC | | |
| exp-ER27-R | GATACCACAGACACCCTAGGTACCTTACTTCTTCTGCTCAGCAAGGTACTTC | | |
| E-27-ck-f | CGTCCACTTGCACAAACACAAAC | | |
| E-27-ck-r | CAGATCAAAGACCTCGAAGTTGGAC | | |
| exp-spHXK1-F | CACAAGACATATCTACAGCAGGTACCATGTCCCTCCACGACGCTTAC | | |
| exp-spHXK1-R | GATACCACAGACACCCTAGGTACCTTAATTCTTGAGATGCTCGGCGAGAATAT | | |
| E-S-ck-f | GCGTCCACTTGCACAAACACAAAC | | |
| E-S-ck-r | GGTTGTCAAAATCGCACCATTCACAG | | |
| exp-TAL-F | CACAAGACATATCTACAGCAGGTACCATGTCTTCCAACTCTCTTGAACAGCT | | |
| exp-TAL-R | GATACCACAGACACCCTAGGTACCCTAAGCGGAGAGCTTGGTCTCAATG | | |
| ETAL-ck-f | CCCATTGATCGAGCCCTAACCC | | |
| ETAL-ck-r | GGTTACAGTGGATACCGTGCTTG | | |
| exp-vhb-F | CACAAGACATATCTACAGCAGGTACCATGCTCGACCAGCAAACCATTAAC | | |
| exp-vhb-R | GATACCACAGACACCCTAGGTACCTTATTCAACGGCTTGAGCGTAGAGATC | | |
| Evhb-ck-f | CGAAACGCACCTAGGACCCT | | |
| Evhb-ck-r | CGAGAACTTCTTTAATGGCACCGAG | | |
| exp-YTH5-F | CACAAGACATATCTACAGCAGGTACCATGTACAAGGTCCATAACCCCTACC | | |
| exp-YTH5-R | GATACCACAGACACCCTAGGTACCTTAGACATGCTCAGTTCCAGGATACTG | | |
| EYTH5-ck-f | CCCATTGATCGAGCCCTAACCC | | |
| EYTH5-ck-r | GAGTCTTTTCGGAACAGGTCCCAG | | |
| G-PGI-F | GAATTAAACACACATCAACAATGGCTCAGTCCTTCACGACC | | |
| G-PGI-R | CTATCTGTTAATTGCCTGCAGTCAAGCGGCCCAAGCCTTGT | | |
| GP-ck-f | CAGCAAGGTGTCCGAACCAAAG | | |
| GP-ck-r | GGTTGAGCTGCTTAATGACCTCC | | |
| T-PFK-F | GAGTATAAGAATCATTCAAAATGATTGAAGGAATCTCCTTTGCGTC | | |
| T-PFK-R | CGTGACATAACTAATTACATGACTAACAAGGATCAATAATACCCTGCTCC | | |
| TefP-ck-f | GGTATTCCTGCCACCATTTCGAAC | | |
| TefP-ck-r | GCAAGGTTTTCAGTATAATGTTACATGCGTAC | | |
| T-GLK-F | GAGTATAAGAATCATTCAAAATGACAATCACTCTGAGTCAGAAGGTTG | | |
| T-GLK-R | CGTGACATAACTAATTACATGACTATGAGTCGTCCGGGTAAGCAG | | |
| TefG-ck-f | GCTATTTCAGATCCAGCTCGATGC | | |
| TefG-ck-r | TAACTCCTTCCTTTTCGGTTAGAGCG | | |
| TEF-GND-F | GAGTATAAGAATCATTCAAAATGACTGACACTTCAAACATCAAGTGAG | | |
| TEF-GND-R | CGTGACATAACTAATTACATGATTAAGCATCGTAAGTGGAAGAAGAAACC | | |
| TG-ck-f | CGAGACATCTTCAAGTACGACGACG | | |
| TG-ck-r | GGTTTTCAGTATAATGTTACATGCGTACACG | | |
| e-spHXK1-F | CTACCGAGGTCTGGTTGGTTTTGACGAGTTTGGCGCCCGTTTTTTCG | | |
| e-spHXK1-R | CACTAGTGTCGACAAGCTTGGGATTTGTCTTAGAGGAACGCATATACAGT | | |
| h-glk1-F | CAAATCCCAAGCTTGTCGACACTAGTGCC | | |
| h-glk1-R | GATGCACCGAATCGTCTGATTGTATGGTATTTCACCGGCGCCATAACTT | | |
| SG-ck-f | TAAGCCTTCTATGATTGTTGGTACTGATGG | | |
| SG-ck-r | ATGCTTAGTTTCGGGTTCCATTGTG | | |
| c2-SG-ck-f | CAGTTGCGATATCAGTCCAGTTGG | | |
| c2-SG-ck-r | CGGAAACGCTCGAATCTGCCG | | |
| g-pgi1-F | GTCTGCGTCTGCTGTTTGTGTCGACGCAGTAGGATGTCCTGCACG | |  |
| g-pgi1-R | GTCGACAAGCTTGTCCGTGCGACTACCGTATTCGAAC | |  |
| h-ER16-g-F | GTAGTCGCACGGACAAGCTTGTCGACACTAGTGCC | |  |
| h-ER16-g-R | GGAGTTTAACTTAACCGGCCAGGGTATTTCACCGGCGCCATAACTTC | |  |
| PE16-ck-f | CCAAGAAGATCCAGCCCGAGC | |  |
| PE16-ck-r | CCCGTTTATATACTGACCGGAACATGG | |  |
| d1-pe16-ck-f | GTAACATTAGTTGTCACGCGGTCC | |  |
| d1-pe16-ck-r | GCACCCCTTTCTCCACACCC | |  |
| t-gnd1-F | GATTTTGCTGCACTAAGACACCAGAGACCGGGTTGGCGGCGC | |  |
| t-gnd1-R | GTCGACAAGCTTGGTTATGCTAGCGGATCCCTTAAGC | |  |
| h-ER25-g-F | CGCTAGCATAACCAAGCTTGTCGACACTAGTGCC | |  |
| h-ER25-g-R | CAGCGCGTTTCTTTTGCAAATGTCGTATTTCACCGGCGCCATAACTTC | |  |
| G-E25-ck-f | CACCTACCAGCTCCTCGATGG | |  |
| G-E25-ck-r | CAGCTTGAATGTTTCTGTGATAATTGGCAT | |  |
| e2-ge25-ck-f | GGACAATGATGAAGCTGCAATGACAC | |  |
| e2-ge25-ck-r | GACACAAATACGCCGCCAACCC | |  |
| t-pfk1-F | TTTTGCTGCACTAAGACACCAGAGACCGGGTTGGCGGCGC | |  |
| t-pfk1-R | CGACAAGCTTGGTTATGCTAGCGGATCCCTTAAGC | |  |
| h-fba1-F | CTAGCATAACCAAGCTTGTCGACACTAGTGCC | |  |
| h-fba1-R | CGTTTCTTTTGCAAATGTCGTATTTCACCGGCGCCATAACTTC | |  |
| PF-ck-f | CCAAGCATTCCTACTGGCACG | |  |
| PF-ck-r | GGACTTTCGCTTAAGGACGTCAG | |  |
| e2-pf-ck-f | GGGTTTGACAGACGCAACAATAGAAAG | |  |
| e2-pf-ck-r | GGACACAAATGCGCCGCCAAC | |  |
| e-ER27-F | GTGCATAATTATAATTCATCACGAGTTTGGCGCCCGTTTTTTCG | |  |
| e-ER27-R | GTCGACAAGCTTGGGATTTGTCTTAGAGGAACGCATATACAGT | |  |
| h-pyp-g-F | CTAAGACAAATCCCAAGCTTGTCGACACTAGTGCC | |  |
| h-pyp-g-R | CTAAAACCTGAATTGAACGAAGCGTATTTCACCGGCGCCATAACTTC | |  |
| E27P-ck-f | CAACTTCGAGGTCTTTGATCTGTCTC | |  |
| E27P-ck-r | CGAAACCTAAAGTATCGGTCAGATAATCGTTAG | |  |
| e3-pe27-ck-f | CTGAATAGAACCCGCATGGAACC | |  |
| e3-pe27-ck-r | CATCATGGGCTCTCGTCCAGC | |  |
| e-tal-F | GAGGAAGTTTGGGGAGAAAGAACGAGTTTGGCGCCCGTTTTTTCG |  |  |
| e-tal-R | GTCGACAAGCTTGGGATTTGTCTTAGAGGAACGCATATACAGT |  |  |
| h-rpi-g-F | CTAAGACAAATCCCAAGCTTGTCGACACTAGTGCC |  |  |
| h-rpi-g-R | GTGTATCCTGTCCCTCTCACTCGTATTTCACCGGCGCCATAACTTC |  |  |
| TR-ck-f | CGACGAGACTTTAACGAGGACCAG |  |  |
| TR-ck-r | CTTCTCGGCAACGTAGACCACG |  |  |
| e4-tr-ck-f | GACCCGCAAGAAAAGACGACC |  |  |
| e4-tr-ck-r | CATCATGGGCTCTCGTCCAGC |  |  |
| p3-THI13-F | GGTGGAGATAACTAGGGAACGGCTAGAGGGGTAGTCGTAAGTTTCATCACG |  |  |
| p3-THI13-R | GTCGACAAGCTTGTCCGTGCGACTACCGTATTCGAAC |  |  |
| h-ER10-g-F | GTAGTCGCACGGACAAGCTTGTCGACACTAGTGCC |  |  |
| h-ER10-g-R | GAATAAAACCCCAGATCCAAGTCCGTATTTCACCGGCGCCATAACTTC |  |  |
| PT-E10-ck-f | GGAAGCCCAAAGATTGATGGCTATTC |  |  |
| PT-E10-ck-r | CTTCCACAGGCCGAATCCGAC |  |  |
| f2-te10-ck-f | CAACTGTCACCAGAGAACCTGC |  |  |
| f2-te10-ck-r | CAAAGGAATCCCCGGAAATCGTG |  |  |
| e-vhb-F | CGAGAATTAGTGTTGATGCTAGAGTTTGGCGCCCGTTTTTTCG |  |  |
| e-vhb-R | GTCGACAAGCTTGGGATTTGTCTTAGAGGAACGCATATACAGT |  |  |
| h-did2-g-F | CTAAGACAAATCCCAAGCTTGTCGACACTAGTGCC |  |  |
| h-did2-g-R | GTGTATAGTAGCTGCGGTAGATGGTATTTCACCGGCGCCATAACTTC |  |  |
| VD-ck-f | CGCTACCGATGACATTCTCGACG |  |  |
| VD-ck-r | GCTCTCAGCTCCACTACAGGAC |  |  |
| f3-vd-ck-f | GCCTACTGCCAATGTTTGTTACTGC |  |  |
| f3-vd-ck-r | CATCATGGGCTCTCGTCCAGC |  |  |
| t-glk1-F | GTCTCCTCTTCACCACCAAAAGAGACCGGGTTGGCGGCGC |  |  |
| t-glk1-R | CGACAAGCTTGGTTATGCTAGCGGATCCCTTAAGC |  |  |
| h-tpi1-g-F | CTAGCATAACCAAGCTTGTCGACACTAGTGCC |  |  |
| h-tpi1-g-R | TATACACAGTTAAATTACAGTATTTCACCGGCGCCATAACTTC |  |  |
| GT-ck-f | CTTAAGGGATCCGCTAGCATAACC |  |  |
| GT-ck-r | CTCGTCAATGGTCTCTCCGATACAG |  |  |
| u3-gt-ck-f | TATGGCGCCGGTGAAATACTGT |  |  |
| u3-gt-ck-r | TCAAACAGACGTTTTGTGCCGGAG |  |  |
| e-yth5-F | GAGCGTTGGATTTTCTGGGAGTTTGGCGCCCGTTTTTTCG |  |  |
| e-yth5-R | CGACAAGCTTGGGATTTGTCTTAGAGGAACGCATATACAGT |  |  |
| h-yth4-g-F | GACAAATCCCAAGCTTGTCGACACTAGTGCC |  |  |
| h-yth4-g-R | CATTTTACGAGGACGGTGTATTTCACCGGCGCCATAACTTC |  |  |
| Y4/5-ck-f | CTCTATGATTACTGTATATGCGTTCCTCTAAGAC |  |  |
| Y4/5-ck-r | GGCTTCACAGCTCCAAACCTTG |  |  |
| c1-y4/5-ck-f | GATACTGCTGCCTTACTGTCGACTG |  |  |
| c1-y4/5-ck-r | CCATCATGGGCTCTCGTCCAGC |  |  |

**Table S6.** Sequences of primers used for Real Time PCR.

| **Primers** | **Sequence 5′→3′** |
| --- | --- |
| Actin-F | TCCCAGTCCTCTTCTCTCGA |
| Actin-R | GGATACCAGCAGCCTCAAGA |
| ZWF1-F | TGGACCATCTGCCTCGAAAT |
| ZWF1-R | AGGCAGCTTGGAGTTCATCT |
| GND1-F | TCTTACGCCCAGGGTTTCAT |
| GND1-R | TCGGTAAGCCTTGGTGATGT |
| TKL1-F | GCTCGGTAACCTCATCACCT |
| TKL1-R | GCCAGATCGTTGTTACCGTC |
| TAL1-F | GAGTCCAAGCACGGTATCCA |
| TAL1-R | GATTCGGCCAACAAAGGGAG |
| RPE1-F | TGAGAGAGCGATACCCCAAC |
| RPE1-R | CGGCGACAATGACATTAGCA |
| RKI1-F | GGGATGCATCGACCAGTACT |
| RKI1-R | TCGGCGACAATCTTTTCCTG |
| ER10-F | GACTACCAGAACGAGCGAGA |
| ER10-R | AGATGTCCTCTCGCTTGACC |
| ER16-F | TTGTCATGACCGCCTTCTCT |
| ER16-R | CCGGGAGAGGTGTTGTACTT |
| ER25-F | ATTCTGGTCACCGCCTACTC |
| ER25-R | TGTTTCTTGGCAATCTCGGC |
| ER27-F | TGCGAGCAAGAACATTGTCC |
| ER27-R | CCAGTTGACAATGAGGGCAG |
| GLK1-F | GCTGTTGGAAAAGACGTCGT |
| GLK1-R | ATCGTCGGATTTAGAGGCGT |
| HXK1-F | GCCTTCGACAATGAGCACAA |
| HXK1-R | GGTCCAGAAGAACCAGACGA |
| PGI1-F | GCTCCCTTTGACCAGTACCT |
| PGI1-R | CAAACAGAACGGGTCCAGTG |
| PFK1-F | TACCCTGCTCTTCGAATCCC |
| PFK1-R | AAGTGTCGGCTCCAAGAGAA |
| FBA1-F | TGTCGACAACTCCAAGCTCT |
| FBA1-R | GTTGCCGACCTTGTAGACAC |
| TPI1-F | GTCAACGGCAAGAACTCTGG |
| TPI1-R | TGTCAACAAACTCGGGCTTG |
| YTH1-F | AGCCAAGGGTGTTGCTCTAT |
| YTH1-R | CAGCCTCCCCAGATGAAGAA |
| YTH3-F | CGGGTTCGCTGCTCTTTATC |
| YTH3-R | CAGAAACAACGACGAAGGCA |
| YTH4-F | CGACGACAACCACAAGACTG |
| YTH4-R | CAGTAGAGCCAGATACCGGG |
| YTH5-F | CATCTTCAAAATGGCCGGCT |
| YTH5-R | AGGGGTCTTCGTCCGATTTT |
| PYP1(S.C.)-F | CCCTGTCATCGTTGTTTCCA |
| PYP1(S.C.)-R | TGTGCATCGATTTCCACTTCA |
| VHB-F | AAACATTGTCAAGCTGGCGT |
| VHB-R | GCATCGCCGAGAACTTCTTT |
| THI13(S.C.)-F | TTCTAGCCGACCCTGTGAAG |
| THI13(S.C.)-R | TCTTCCAGTCACGGTGAACA |
| DID2-F | GGACATTGCTGCTCTGTACG |
| DID2-R | TGATGCCACAGACTCCACTC |

**Table S7.** Sequences of *Vhb* and *SpHXK1*.

| **Genes** | **Sequence 5′→3′** |
| --- | --- |
| *Vhb* | ATGCTCGACCAGCAAACCATTAACATCATCAAAGCCACTGTTCCTGTTCTCAAGGAGCATGGCGTTACCATTACCACCACTTTTTATAAAAACCTCTTTGCCAAACACCCTGAAGTTCGACCTCTCTTTGATATGGGACGACAAGAATCTCTCGAACAGCCTAAGGCTCTCGCCATGACCGTTCTCGCCGCTGCCCAAAACATTGAAAATCTCCCTGCTATTCTCCCTGCCGTCAAAAAAATTGCTGTCAAACATTGTCAAGCTGGCGTGGCTGCTGCCCATTATCCCATTGTCGGTCAAGAACTCCTCGGTGCCATTAAAGAAGTTCTCGGCGATGCCGCTACCGATGACATTCTCGACGCCTGGGGCAAGGCTTATGGCGTGATTGCTGATGTGTTTATTCAAGTGGAAGCTGATCTCTACGCTCAAGCCGTTGAATAA |
| *SpHXK1* | ATGTCCCTCCACGACGCTTACCATTGGCCTTCTCGAACTCCTTCCCGAAAGGGTTCTAATATCAAACTCAACAAAACTCTCCAAGATCATCTCGATGAACTGGAAGAACAATTCACCATTCCCACTGAACTCCTCCATCGAGTTACCGATCGATTTGTTTCTGAACTCTACAAGGGCCTCACCACCAACCCCGGTGATGTTCCTATGGTCCCCACTTGGATCATTGGTACTCCTGATGGCAATGAGCATGGCTCTTATCTCGCTCTCGATCTCGGTGGTACTAACCTCCGAGTTTGTGCTGTTGAGGTTCAAGGCAACGGTAAATTCGACATTACTCAATCCAAATACCGACTCCCTCAAGAACTCAAAGTTGGCACCCGAGAGGCCCTCTTTGATTACATTGCCGACTGTATCAAGAAATTTGTGGAAGAGGTTCACCCTGGTAAATCCCAAAATCTCGAAATTGGTTTCACCTTTTCTTACCCCTGTGTTCAACGATCCATTAACGATGCTTCTCTCGTTGCCTGGACTAAGGGCTTTGATATTGATGGCGTTGAGGGTGAATCCGTTGGTCCTCTCCTCTCTGCTGCCCTCAAGCGAGTTGGCTGTAACAACGTTCGACTCAATGCCATTCTCTCCGATACTACTGGTACTCTCGTTGCTTCCAACTATGCCTCCCCTGGTACTGAGATTGGTGTCATCTTTGGAACTGGATGTAATGCTTGTTACATTGAAAAGTTCTCTGAAATTCCTAAGCTCCATAAGTATGACTTCCCTGAAGATATGAACATGATCATCAACTGTGAATGGTGCGATTTTGACAACCAGCATGTTGTCCTCCCTCGAACCAAATACGATGTTGCTATTGATGAAGAGTCTCCCCGACCCGGTCTCCAAACCTACGAGAAAATGATTGCTGGATGCTATCTCGGTGATATCCTCCGACGAATCCTCCTCGACCTCTATGAACAGGGAGCCCTCTTTAACGGTCAGGACGTTACCAAGATTCGAGACCCCCTCGCCATGGATACCTCTGTGCTCTCCGCTATTGAAGTTGACCCCTTTGAGAACCTCGATGAAACTCAAACCCTCTTTGAGGAAACCTATGGTCTCAAGACCACCGAAGAAGAGCGACAATTCATTCGACGAGCTTGCGAACTCATTGGAACTCGATCTGCCCGACTCTCTGCCTGTGGTGTTTGCGCCCTCGTTCGAAAAATGAATAAGCCTTCTATGATTGTTGGTACTGATGGTTCCGTCTACAACCTCTACCCTCGATTTAAGGATCGACTCGCTCAAGCCTTTAAGGATATCCTCGGTGAGGAAATTGGCTCCAAAGTTGTTACCATCCCCGCCGAAGACGGTTCCGGCGTTGGTGCTGCTCTCGTCTCCGCTCTCGAAGCCAAAGGCAAGGCCCTCACTTCTGATATTCTCGCCGAGCATCTCAAGAATTAA |

**Table S8.** Relative growth rate and glucose uptake rate (Q_GLU_) of the engineered *Y.lipolytica* strains.

| **Strains**  **(description)** | **Relative growth**  **rate (h^-1^)** | **Q_GLU_ [g/(L·h)]** | **Time h** |
| --- | --- | --- | --- |
| Ylxs01 (the parental strain) | 0.055 | 3.97 | 78 |
| Ylxs02 (intA:: P_tef_-*ZWF1*-T_xpr2_) | 0.041 | 3.69 | 84 |
| Ylxs03 (intA:: P_hp4d_-*ZWF1*-T_xpr2_) | 0.036 | 3.23 | 96 |
| Ylxs04 (intA:: P_hp4d_-*GND1*-T_xpr2_) | 0.043 | 3.69 | 84 |
| Ylxs17 (intA:: P_hp4d_-*YHT1*-T_xpr2_) | 0.057 | 4.31 | 72 |
| Ylxs18 (intA:: P_hp4d_-*YHT3*-T_xpr2_) | 0.054 | 4.56 | 68 |
| Ylxs19 (intA:: P_hp4d_-*YHT4*-T_xpr2_) | 0.056 | 4.31 | 72 |
| Ylxs20 (intA:: P_hp4d_-*YHT5*-T_xpr2_) | 0.055 | 4.31 | 72 |
| Ylxs21 (intA:: P_hp4d_-*GLK1*-T_xpr2_) | 0.054 | 3.97 | 78 |
| Ylxs22 (intA:: P_hp4d_-*HXK1*-T_xpr2_) | 0.056 | 4.08 | 76 |
| Ylxs23 (intA:: P_hp4d_-*SpHXK1*-T_xpr2_) | 0.041 | 3.69 | 84 |
| Ylxs48 (the final engineered strain) | 0.054 | 6.74 | 46 |

The engineered *Y.lipolytica* strains were cultivated in 20 mL YPNP medium with 310 g/L glucose at 30 °C, and 220 rpm in 250 mL baffled flasks. The relative growth rate was calculated by (ln OD_600e_ - ln OD_600i_)/12, where OD_600e_ and OD_600i_ are the biomass at 24 h and 12 h respectively. The fermentation ended at different time point when the carbon source was completely consumed and glucose uptake rate (Q_GLU_) was calculated.

**Table S9.** Key performance indicators of erythritol synthesis by the parental and engineered *Y.lipolytica* strains under 100 L batch and glucose fed-batch conditions.

| **Strains** | **Type** | **P_ERY_**  **(g/L)** | **Y_ERY_**  **(g/g GLU)** | **Q_ERY_**  **[g/(L⋅h)]** | **T**  **(h)** | **Q_GLU_**  **[g/(L⋅h)]** | **R_ERY_**  **[g/(g DCW·h)]** | **CA**  **g/L** |
| --- | --- | --- | --- | --- | --- | --- | --- | --- |
| Ylxs01 | batch | 178.85 | 0.57 | 2.63 | 68 | 4.56 | 0.26 | 8.35 |
| Ylxs48 | batch | 211.07 | 0.69 | 4.91 | 43 | 7.21 | 0.53 | 4.83 |
| Ylxs48 | fed-batch | 322.21 | 0.71 | 4.35 | 74 | 5.73 | 0.47 | 4.18 |

P_ERY_: erythritol production titer; Q_ERY_: erythritol volumetric productivity; Y_ERY_: erythritol yield; T: the fermentation time when carbon source was completely consumed; Q_GLU_: glucose consumption rate; R_ERY_: the specific erythritol productivity based on the cell biomass; The *Y.lipolytica* strains were cultivated in 50 L YPNP medium with 310 g/L glucose at 30 °C, and 500 rpm in the 100 L bioreactor under batch fermentation. Initial 40 L of YPNP medium supplemented with 310 g/L glucose was used under the fed-batch fermentation. More glucose (3200 g, 3200 g, 2400 g) was added to the bioreactor in triplicate at 4 L,4 L and 2 L each. The fermentation ended at different time point when the carbon source was completely consumed and citric acid (CA) titer were detected by HPLC.

**Table S10.** Distribution of glucose consumption by the parental and engineered *Y.lipolytica* strains under 100 L batch and glucose fed-batch conditions.

| **Strains** | **Type** | **M_TIG_**  **(g)** | **M_ARG_**  **(g)** | **M_PPG_**  **(g)** | **M_CAG_**  **(g)** | **M_DCW_**  **(g)** | **M_ERYT_**  **(g)** | **M_ERYA_**  **(g)** | **M_OTHER_**  **(g)** |
| --- | --- | --- | --- | --- | --- | --- | --- | --- | --- |
| Ylxs01 | batch | 15500 | 3652.56 | 1224.74 | 391.41 | 516 | 9877.21 | 8942.5 | 934.71 |
| Ylxs48 | batch | 15500 | 2483.91 | 1342.51 | 226.41 | 464 | 11166.22 | 10553.5 | 612.72 |
| Ylxs48 | fed-batch | 21200 | 3413.16 | 2027.74 | 195.94 | 425 | 15390.46 | 15143.87 | 246.59 |

M_TIG_: total initial glucose weight; M_ARG_: glucose consumed by aerobic respiration; M_PPG_: glucose consumed by the pentose phosphate pathway; M_CAG_: glucose consumed to produce citric acid; M_DCW_: dry weight of biomass; M_ERYT_: the theoretical weight of erythritol produced; M_ERYA_: the actual weight of erythritol produced; M_OTHER_: glucose consumption in other possible pathways and measurement errors; It is assumed that CO_2_ production comes from the cellular aerobic respiration (C_6_H_12_O_6_+6O_2_→6CO_2_+6H_2_O) and pentose phosphate pathways (C_6_H_12_O_6_+H_2_O+2NADP^+^→ C_5_H_10_O_5_+CO_2_+2NADPH+2H^+^), and the O_2_ consumed is used for aerobic respiration. The CO_2_ production and O_2_ consumption (mmol/L) are obtained by integrating the exhaust analysis data. The *Y.lipolytica* strains were cultivated in 50 L YPNP medium with 310 g/L glucose at 30 °C, and 500 rpm in the 100 L bioreactor under batch fermentation. Initial 40 L of YPNP medium supplemented with 310 g/L glucose was used under the fed-batch fermentation. More glucose (3200 g, 3200 g, 2400 g) was added to the bioreactor in triplicate at 4 L,4 L and 2 L each. The fermentation ended at different time point when the carbon source was completely consumed and citric acid (CA) titer were detected by HPLC.


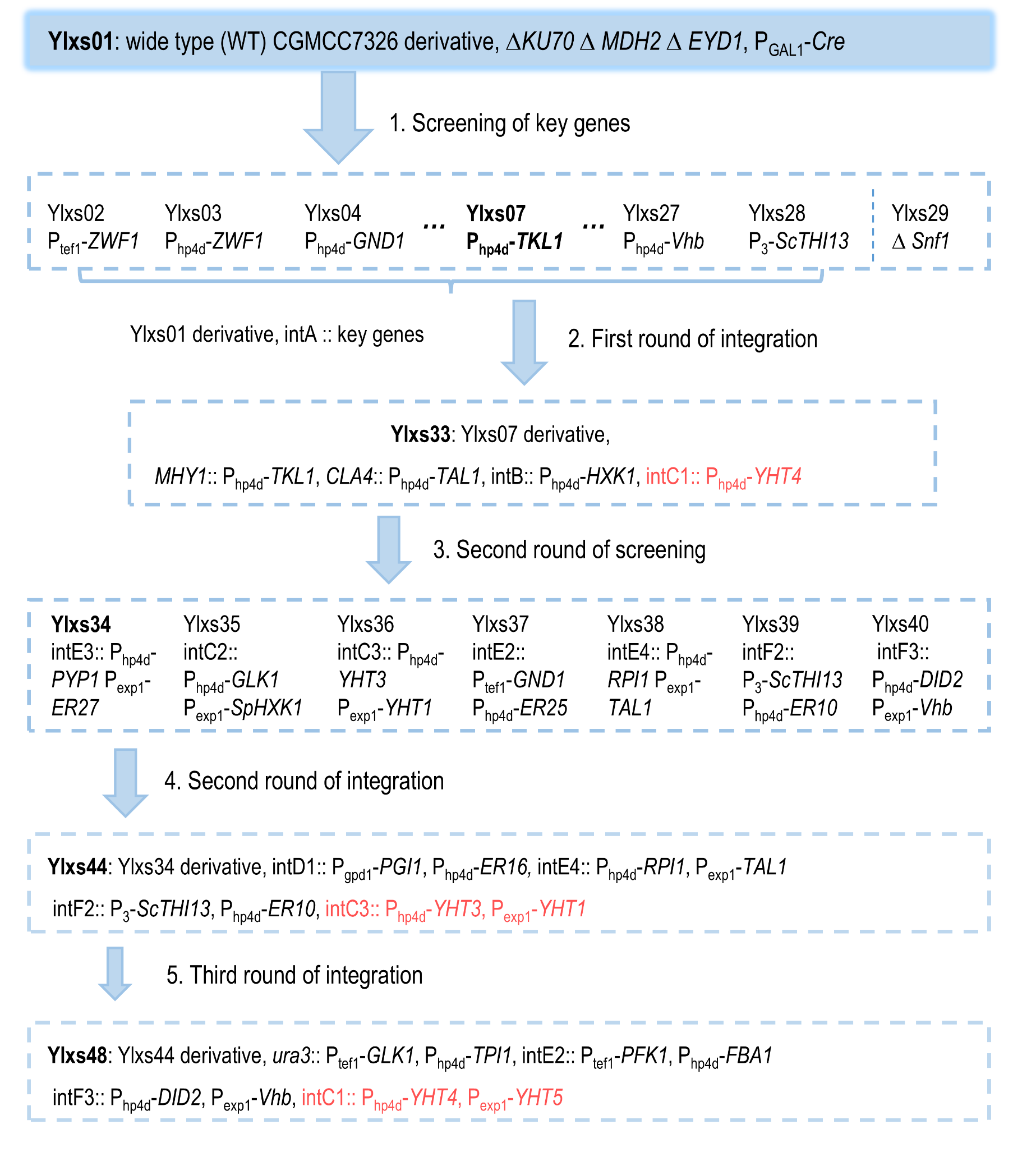


**Fig. S1.** Schematic diagram of strain construction process.


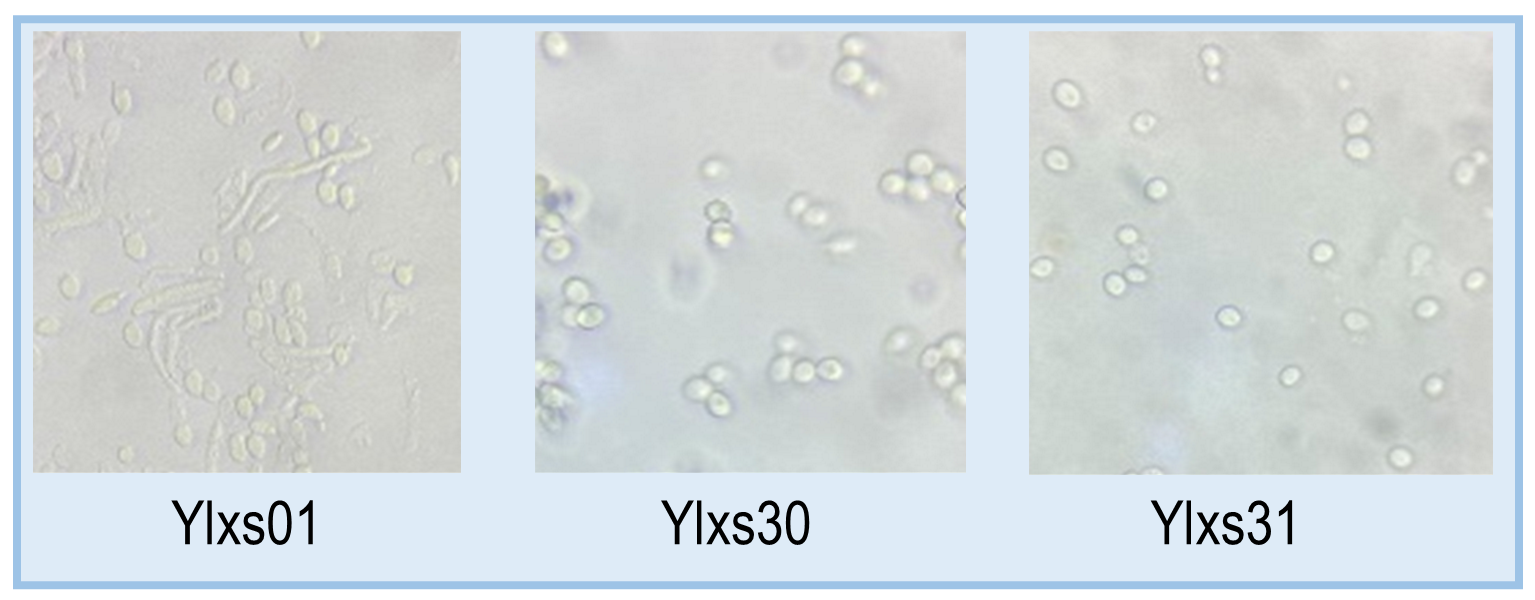


**Figure S2.** The microscopy images of the control strain Ylxs01, Ylxs30 (Ylxs07 derivative, *MHY1*:: P_hp4d_-*TKL1*-T_xpr2_), and Ylxs31 (Ylxs30 derivative, *CLA4*:: P_hp4d_-*TAL1*-T_xpr2_) grown on YNB plates for 4 days. The control strain Ylxs01 contains fungi-like cells, while Ylxs30 and Ylxs31 only contain yeast-like cells.


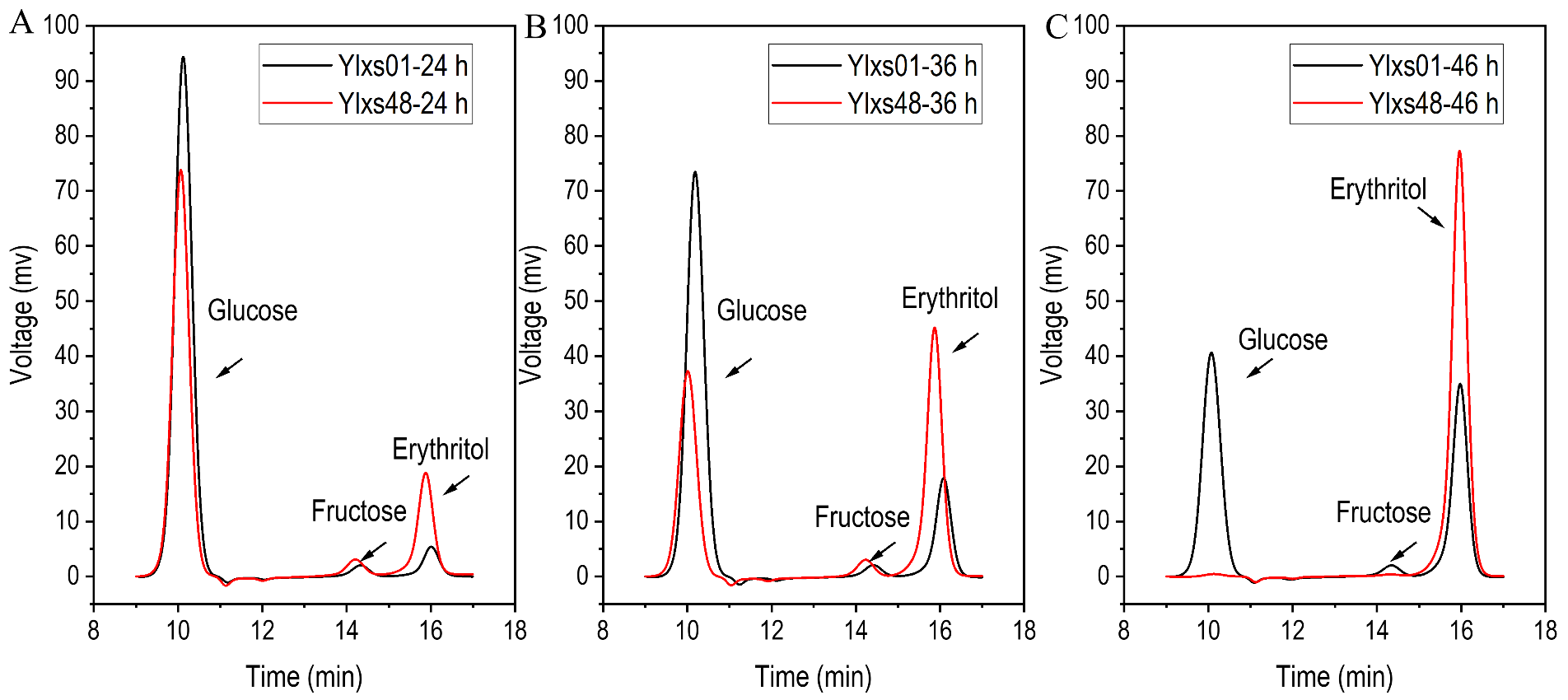


**Figure S3.** Chromatographic diagram of substrates and products analysis of the control strain Ylxs01 and the engineered *Y.lipolytica* Ylxs48. The strains was cultivated in 1.5 L YPNP medium supplemented with glucose (310 g/L) at 30 °C, and 700 rpm under 3 L bioreactors for 24 h (A), 36 h (B) and 46 h (C).


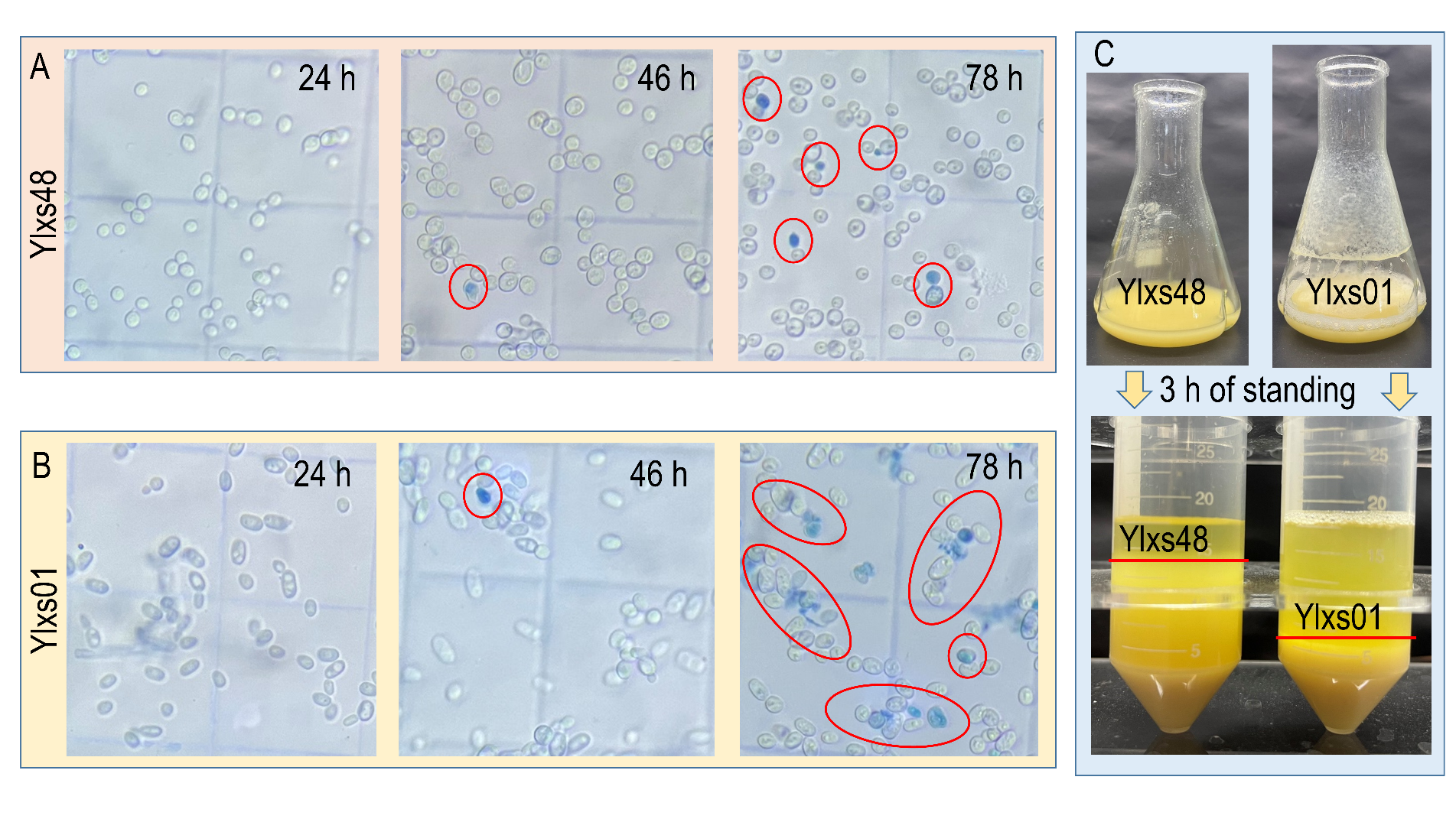


**Figure S4.** Comparison of cell activity/viability of the control strain Ylxs01 and the engineered *Y.lipolytica* Ylxs48 at different fermentation times. (A) The microscopy images of the Ylxs48 during erythritol production in 250 mL baffle shakers containing 20 mL of YPNP medium supplemented with 310 g/L glucose (24-46-78 h). (B) The microscopy images of the Ylxs01 during erythritol production in 250 mL baffle shakers containing 20 mL of YPNP medium supplemented with 310 g/L glucose (24-46-78 h). (C) The images of the strains Ylxs01 and Ylxs48 stand for 3 h after fermentation.


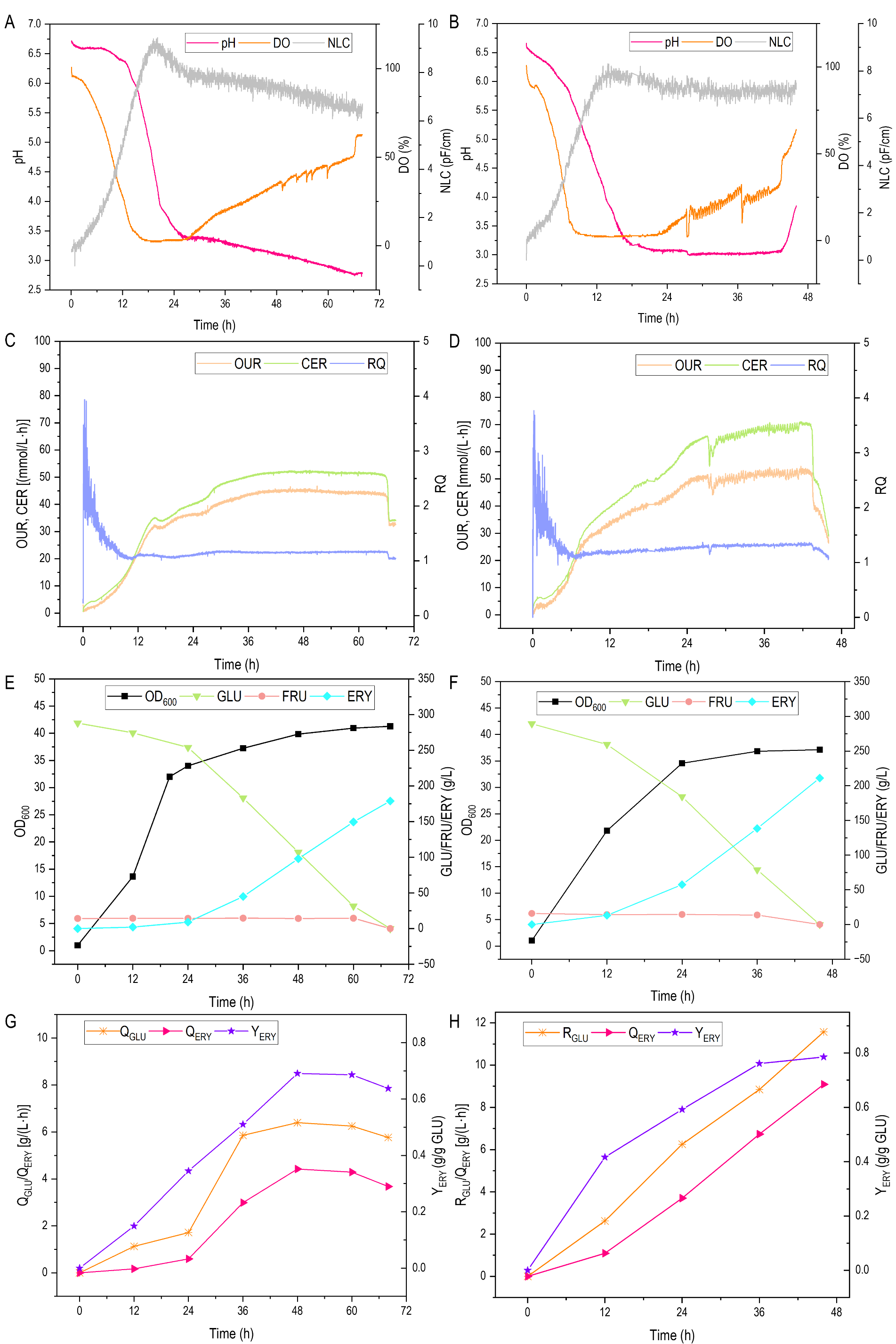


**Figure S5.** Schematic diagram of the glucose batch fermentation process of the parental strain Ylxs01 and the engineered strain Ylxs48 in the 100 L bioreactor. Time course effects on cell growth (Number of living cells, NLC), DO and pH changes of the parental strain Ylxs01 (A) and the engineered strain Ylxs48 (B). Time course effects on oxygen uptake rate (OUR), carbon dioxide evolution rate (CER) and respiration quotient (RQ) changes of the parental strain Ylxs01 (C) and the engineered strain Ylxs48 (D).Time course effects on cell growth (OD_600_), glucose, fructose and erythritol titer changes of the parental strain Ylxs01 (E) and the engineered strain Ylxs48 (F). Time course effects on glucose consumption rate (R_GLU_), productivity (Q_ERY_) and Yield (Y_ERY_) under the batch fermentation by the parental strain Ylxs01 (G) and the engineered *Y. lipolytica* Ylxs48 (H) in the 100 L bioreactor containing 50 L of YPNP medium supplemented with 310 g/L glucose.


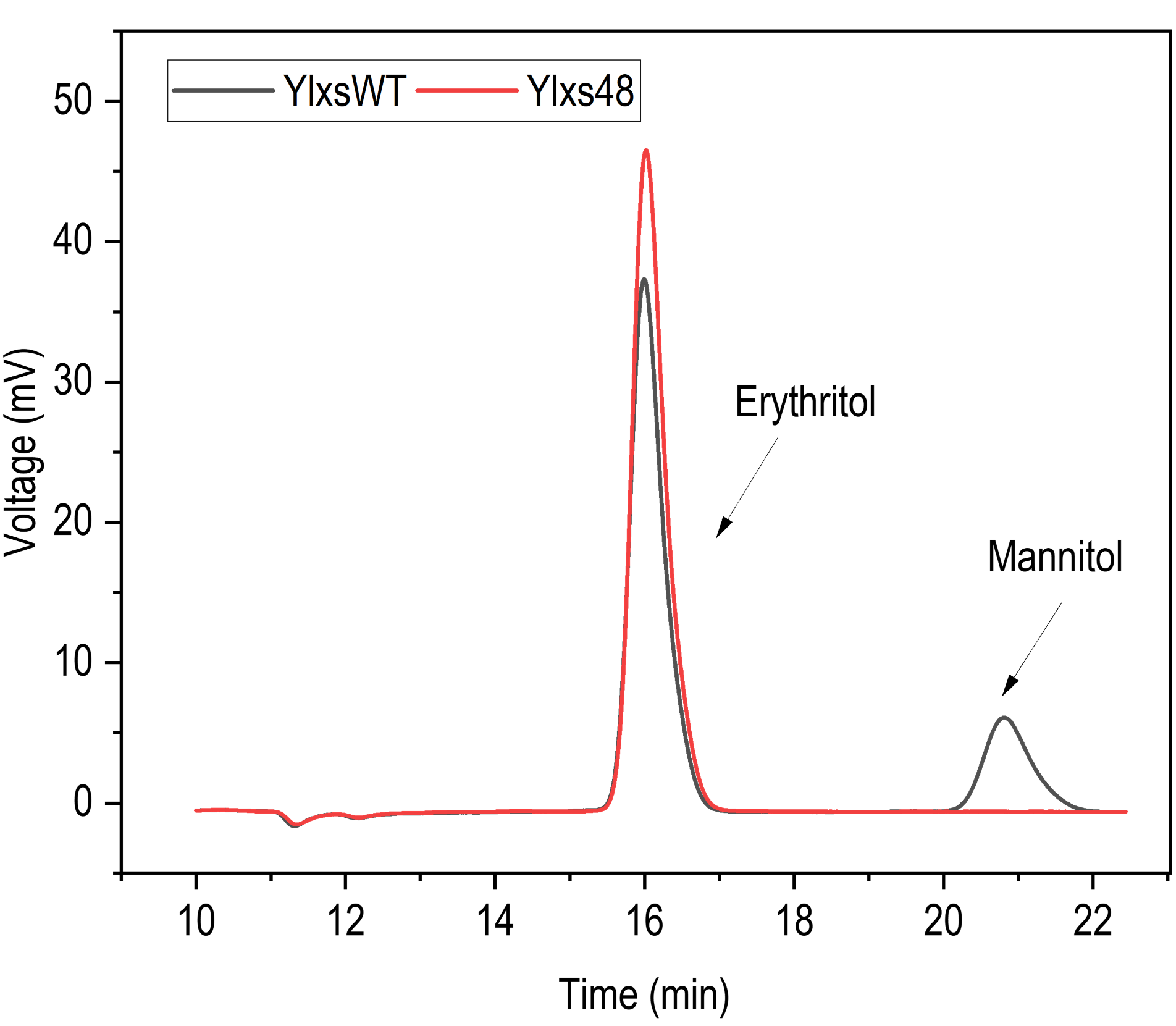


**Figure S6.** Chromatographic diagram of polyol products analysis of the control strain YlxsWT and the engineered *Y.lipolytica* Ylxs48. The strains was cultivated in 20 mL YPNP medium supplemented with glucose (100 g/L) at 30 °C, and 220 rpm in 250 mL baffled flasks for 36 h.

**Methods:** Construction of homologous recombinant integrative plasmids.

**pSnf1-*guaB*:** Snf1-up (upstream seq) and Snf1-dw (downstream seq) were amplified from *Y. lipolytica* genome with primers pair snf1-up-F/snf1-up-R and snf1-dw-F/snf1-dw-R separately and then were cloned into *Eco*RI loci of the plasmid pXS*-guaB* (1)*,* yielding pSnf1-updw. The marker was amplified with primers pair snf-guaB-F/snf-guaB-R from pXS*-guaB*, cloned into *Nde*I loci of pSnf1-updw to generate pSnf1-*guaB*.

**pTef1-*guaB*:** The tef1 fragment was amplified from *Y. lipolytica* genome (1 kb upstream of the YALI0_C09141g) with primers pair TEF-F/TEF-R. The cyc1 fragment was amplified from pXS-*guaB* with primers pair CYC1-F/CYC1-R. Then, the two fragments were cloned into pXS-*guaB* between *Sal*I and *Afl*II, yielding pTef1-*guaB*.

**pGpd1-*guaB*:** The gpd1 fragment was amplified from *Y. lipolytica* genome (1 kb upstream of the YALI0_C06369g) with primers pair GPD-F/GPD-R and then was cloned into pXS-*guaB* between *Sal*I and *Kpn*I, yielding pGpd1-*guaB*.

**pTef1-*ZWF1*:** The tef1 and *ZWF1* fragments were amplified from *Y. lipolytica* genome (1 kb upstream of the YALI0_C09141g and 1937 bp of the YALI0_E22649g) with primers pair TEF-Z-F/TEF-Z-R and T-ZWF1-F/T-ZWF1-R separately, and then were cloned into pIntA-*guaB* between *Hin*dIII and *Kpn*I, yielding pTef1-*ZWF1*.

**pIntA-*ScTHI13*:** The p3 fragment was amplified from *Y. lipolytica* genome (1 kb upstream of the YALI0_A09768g) with primers pair P3-F/P3-R. The gene *ScTHI13* was amplified from *Saccharomyces cerevisiae* genome with primers pair P3-THI13-F/P3-THI13-R. Then, the two fragments were cloned into pXS-*guaB* between *Hin*dIII and *Kpn*I, yielding pIntA-*ScTHI13*.

**pIntC2-updw:** intC2-up and intC2-dw were amplified from *Y. lipolytica* genome intC2 loci with primers pair C2-up-F/C2-up-R and C2-dw-F/C2-dw-R separately and then were cloned into *Eco*RI loci of the plasmid pXS-*guaB*, yielding pIntC2-updw. The pIntD1- updw, pIntE2- updw, pIntE3- updw, pIntE4- updw, pF2- updw and pF3- updw were constructed with similar process.

**pIntA-*PYP1*/*Vhb***/***SpHXK1*:** The gene *PYP1* was amplified from *Saccharomyces cerevisiae* genome with primers pair PYP1-F/PYP1-R and cloned into *Kpn*I loci of pIntA-*guaB*, yielding pIntA-*PYP1*. *Vhb* from *Vitreoscilla stercoraria* and *SpHXK1* from *Schizosaccharomyces pombe* were artificially synthesized by GENERAL BIOL (Table S7) and cloned into *Kpn*I loci +of pIntA-*guaB* separately, yielding pIntA-*Vhb* and pIntA- *SpHXK1*.

**pIntA-*ZWF1*:** *ZWF1* was amplified from *Y. lipolytica* genome with primers pair ZWF1-F/ZWF1-R and cloned into *Kpn*I loci of pIntA-*guaB*, yielding pIntA-*ZWF1.* The pIntA-*GND1*, pIntA-*RPE1*, pIntA-*RPI1*, pIntA-*TKL1*, pIntA-*TAL1*, pIntA-*PGI1*, pIntA-*PFK1*, pIntA-*FBA1*, pIntA-*TPI1*, pIntA-*ER10*, pIntA-*ER16*, pIntA-*ER25*, pIntA-*ER27*, pIntA-*YHT1*, pIntA-*YHT3*, pIntA-*YHT4*, pIntA-*YHT5*, pIntA-*GLK1*, pIntA-*HXK1*, pIntA-*AMPD* and pIntA-*DID2* were constructed with similar process*.*

**pMhy1-*TKL1*:** The gene *TKL1* was amplified from *Y. lipolytica* genome with primers pair TKL1-F/TKL1-R and cloned into *Kpn*I loci of pMhy1-*guaB*, yielding pMhy1-*TKL1*. The pCla4-*TAL1*, pIntB-*HXK1,* and pIntC1-*YHT4* were constructed with a similar process*.*

**pIntC2-*GLK1/SpHXK1*:** The gene *SpHXK1* was amplified from pIntA-*SpHXK1* with primers pair exp-spHXK1-F/exp-spHXK1-R and cloned into *Kpn*I loci of pExp1-*guaB*, yielding pExp1-*SpHXK1*. The P_exp1_-*SpHXK1*-T_lip2_ and P_hp4d_-*GLK1*-T_xpr2_-*guaB* cassettes were amplified with primers pair e-spHXK1-F/e-spHXK1-R and h-glk1-F/h-glk1-R separately and then were cloned into *Bam*HI loci of the plasmid pIntC2-updw, yielding pIntC2-*GLK1/SpHXK1*. The pIntF3-*DID2*/*Vhb* was constructed with a similar process.

**pIntD1-*PGI1/ER16*:** The gene *PGI1* was amplified from *Y. lipolytica* genome with primers pair G-PGI-F/G-PGI-R and cloned into *Kpn*I loci of pGpd1-*guaB*, yielding pGpd1-*PGI1*. The P_gpd1_-*PGI1*-T_xpr2_ and P_hp4d_-*ER16*-T_xpr2_-*guaB* cassettes were amplified with primers pair g-pgi1-F/g-pgi1-R and h-ER16-g-F/h-ER16-g-R separately and then were cloned into *Bam*HI loci of the plasmid pIntD1-updw, yielding pIntD1-*PGI1/ER16*.

**pIntF2-*ScTHI13/ER10*:** The P_3_-*ScTHI13*-T_xpr2_ and P_hp4d_-*ER10*-T_xpr2_-*guaB* cassettes were amplified with primers pair p3-THI13-F/p3-THI13-R and h-ER10-g-F/h-ER10-g-R separately and then were cloned into *Bam*HI loci of the plasmid pIntF2-updw, yielding pIntF2-*ScTHI13/ER10*.

**pIntE3-*PYP1*/*ER27*:** The gene *ER27* was amplified from *Y. lipolytica* genome with primers pair exp-ER27-F/exp-ER27-R and cloned into *Kpn*I loci of pExp1-*guaB*, yielding pExp1-*ER27*. The P_exp1_-*ER27*-T_lip2_ and P_hp4d_-*PYP1*-T_xpr2_-*guaB* cassettes were amplified with primers pair e-ER27-F/e-ER27-R and h-pyp-g-F/h-pyp-g-R separately and then were cloned into *Bam*HI loci of the plasmid pIntE3-updw, yielding pIntE3-*PYP1*/*ER27*. The pIntE4-*RPI1/TAL1* and pIntC1-*YHT4/5* were constructed with a similar process.

**pIntE2-*GND1/ER25*:** The gene *GND1* was amplified from *Y. lipolytica* genome with primers pair TEF-GND-F/TEF-GND-R and cloned into *Kpn*I loci of pTef1-*guaB*, yielding pTef1-*GND1*. The P_tef1_-*GND1*-T_cyc1_ and P_hp4d_-*ER25*-T_xpr2_-*guaB* cassettes were amplified with primers pair t-gnd1-F/t-gnd1-R and h-ER25-g-F/h-ER25-g-R separately and then were cloned into *Hin*dIII loci of the plasmid pIntE2-updw, yielding pIntE2- *GND1/ER25*. The pIntE2-*PFK1/FBA1* and pUra3-*GLK1/TPI1* were constructed with a similar process.

All plasmid constructions were performed by Gibson assembly.

**Supplementary references**

1. Wang N, Chi P, Zou Y, Xu Y, Xu S, Bilal M, Fickers P, Cheng H. 2020. Metabolic engineering of *Yarrowia lipolytica* for thermoresistance and enhanced erythritol productivity. Biotechnol Biofuels 13:176.

2. Xu S, Zhang X, Zhang Y, Li Q, Ji L, Cheng H. 2023. Concomitant production of erythritol and beta-carotene by engineered *Yarrowia lipolytica*. J Agric Food Chem 71:11567-11578.
